# Imaging-based representation and stratification of intra-tumor heterogeneity via tree-edit distance

**DOI:** 10.1038/s41598-022-23752-2

**Published:** 2022-11-15

**Authors:** Lara Cavinato, Matteo Pegoraro, Alessandra Ragni, Martina Sollini, Paola Anna Erba, Francesca Ieva

**Affiliations:** 1 MOX Lab, Department of Mathematics, Politecnico di Milano, Milan, 20133 Italy; 2 Department of Biomedical Sciences, Humanitas University, Pieve Emanuele, 20090 Italy; 3 IRCCS Humanitas Research Hospital, Rozzano, 20089 Italy; 4 Regional Center of Nuclear Medicine, Department of Translational Research and Advanced Technologies in Medicine and Surgery, University of Pisa, Pisa, 56126 Italy; 5 Medical Imaging Center, University of Groningen, University Medical Center Groningen, Groningen, The Netherlands; 6 Human Technopole, Health Data Science Center, Milan, 20157 Italy

**Keywords:** Cancer imaging, Cancer models, Metastasis, Tumour heterogeneity, Prognostic markers, Applied mathematics, Statistics

## Abstract

Personalized medicine is the future of medical practice. In oncology, tumor heterogeneity assessment represents a pivotal step for effective treatment planning and prognosis prediction. Despite new procedures for DNA sequencing and analysis, non-invasive methods for tumor characterization are needed to impact on daily routine. On purpose, imaging texture analysis is rapidly scaling, holding the promise to surrogate histopathological assessment of tumor lesions. In this work, we propose a tree-based representation strategy for describing intra-tumor heterogeneity of patients affected by metastatic cancer. We leverage radiomics information extracted from PET/CT imaging and we provide an exhaustive and easily readable summary of the disease spreading. We exploit this novel patient representation to perform cancer subtyping according to hierarchical clustering technique. To this purpose, a new heterogeneity-based distance between trees is defined and applied to a case study of prostate cancer. Clusters interpretation is explored in terms of concordance with severity status, tumor burden and biological characteristics. Results are promising, as the proposed method outperforms current literature approaches. Ultimately, the proposed method draws a general analysis framework that would allow to extract knowledge from daily acquired imaging data of patients and provide insights for effective treatment planning.

## Introduction

The current paradigm shifting of modern medical practice sinks its root in providing personalized treatments and improving therapy outcomes. Huge strides have been made in oncology with the uprising of quantitative imaging techniques and new procedures for DNA sequencing and analysis that allow an extensive characterization of cancer subtypes. In particular, recent research has investigated the main causes of cancer progression, resistance to therapy and late recurrence. Among these, tumor heterogeneity has gained special interest and has been recognized to play a crucial role^1^: defined as complex genetic, epigenetic and protein modifications that can be found within the same patient’s disease, tumor heterogeneity behaves as a driver for phenotypic selection. According to Stanta and Bonin and y Cajal et al.^2,3^, different types of tumor manifestation may exist as a response to microenvironmental and external changing, differing between primary tumor and proximal and distant metastases. As a result, certain tumor phenotypes properly respond to therapies and others become resistant clones, leading to treatments ineffectiveness and cancer progression. Pertinently, detecting at baseline which phenotype will respond and which will not—known as *prognostic cancer subtyping*—represents a pivotal step in personalized medicine.

Although recent findings about heterogeneity suggest that therapy would be improved if guided by the analysis of both primary and metastatic tissues—such as lymph nodes^4^—, clinical practice usually relies on primary tumor biomarkers for prognosis definition and treatment planning. Thus, baseline assessment emerges altered by the understimation of intra-tumor heterogeneity which behaves as confounding factor in pre-treatment clinical-pathological prognosis, leading to poor survival rates^5^. This misalignment between research evidence and clinical practice seems mostly due to the lack of non-invasive methods for heterogeneity quantification. Accordingly, current prognostic cancer subtyping cannot be translated into daily clinical practice and therapeutic guidelines.

Over the last two decades, the texture analysis of digital images—such as Magnetic Resonance Imaging (MRI) and Positron Emission Tomography/Computer Tomography (PET/CT)—has arisen as a valuable non-invasive proxy for biological assessment of tumors, eventually growing in a discipline of its own, namely radiomics^6^. Specifically, macroscopic appearance of tumors has been acknowledged as a valid tool for guiding clinical decisions in the definition of disease severity and treatment planning. Broadly speaking, image texture analysis consists of extracting descriptors of spatial variation of voxel grey-scale and intensity within the image Volumes Of Interest (VOI), i.e., the tumor lesions. Under the name of radiomic features, such textural descriptors form a high dimensional vector embedding of the VOI and may provide a non-invasive assessment of tumor appearance from routinely acquired imaging studies^7^. These features are indeed supposed to supply additional predictive and prognostic information, ready to use to postulate the underlying biological mechanisms of disease progression in clinical routine^8^. Accordingly, the dissimilarity in the appearance of different lesions, therefore in their texture descriptions, can be regarded as *radiological* heterogeneity, which can be easily quantified and leveraged in the daily practice.

Despite the increasing interest in tumor heterogeneity, imaging-guided therapy currently employs biomarkers for tumor burden that stem from the characterization of the primary tumor, the bigger lesion (often coinciding with the hottest lesion) or the mean lesions’ profile. Only recently few radiomics-based approaches have been suggested—for prognosis, treatment outcome and survival prediction—which consider the multi-lesion disease in a comprehensive way. In particular, several researchers^9–11^ proposed different segmentation strategies for feature extraction from patient level VOIs, while Cottereau et al.^12^ evaluated the predictive power of several indicators reflecting the spatial distributions of malignant *foci* spread throughout the whole body. A number of *dissemination* features have been explored and reviewed: the number of lesions, the euclidean distance between crucial or predominant bulks, the largest value of the pairwise sum of the physical distances between lesions, etc. Stemming from a similar idea, Cavinato et al.^13^ proposed a similarity metric for comparing lesions’ texture descriptions, defining intra-patient heterogeneity as the normalized average of pairwise distances between lesions’ radiomic vectors. This similarity over patient’s lesions description has thus been suggested as functional, rather than spatial, dispersion index for tumor burden and disease severeness, with promising results in Hodgkin Lymphoma^14^ and Prostate Cancer^15^. Preliminary results represent an insightful starting point in the debate around the proper definition of heterogeneous disease.

In this work, motivated by the need to embed tumor heterogeneity quantification into patients’ clinical pathway planning, we propose a novel way for modeling intra-patient tumor heterogeneity in a non-invasive way, leveraging the radiomic framework. Specifically, we perform dimensionality reduction on radiomic vectors, as to remove redundancy and collinearity while preserving the multi-view nature of the texture description. Reduced vectors of peer lesions within the same tumor are then compared via pairwise distances. Representing the patient via the pairwise distance matrix of its lesions makes it laborious to compare patients with different numbers of lesions. For this reason, upon lesions’ distance matrix, we build a dendrogram, which hierarchically aggregates peer lesions in a unique combinatorial object. This object-oriented representation summarizes the multi-lesion disease and highlights the relationship among lesions, basing on similarities in their imaging characteristics. In fact, lesions are not independent as they are statistically and semantically connected to the patient they belong to. Accordingly, such relationship shapes and influences the structure of the dendrogram associated to the patient. We then exploit the tree-based patient representation to cluster cancer subtypes according to their imaging heterogeneity. To do so, we define a new *ad hoc* distance between trees. To validate the method, we test the whole pipeline on a dataset of patients affected by metastatic Prostate Cancer (PCa), evaluating the descriptive and stratification performance in terms of disease severeness and outcomes. We associate imaging subtypes to clinically relevant information within and beyond clinical surrogates, with the goal of eventually supporting therapy decisions wherein actions regarding active surveillance, mild treatment or intensified therapy are devised and taken^1^.

## Results

### Case study: prostate cancer

Within the personalized medicine framework, Prostate cancer (PCa) is a striking example of the need to exploit an insightful prognostic cancer subtyping for treatment planning. In fact, even if recent studies have reported a decreasing pattern of overall PCa incidence, Culp et al.^16^ and Siegel et al.^17^ recorded an alarming mortality rate due to an increasing trend of distant stage metastatic disease, even in developed countries. Moreover, the role of imaging-guided therapy for PCa has revealed to be very promising and is consistently spreading in daily practice^18^. Despite these facts, clinical guidelines still rely on primary tumor biomarkers. Besides, very limited methods have been proposed for reliably assessing and quantifying multi-lesion heterogeneity information within the same patient from an imaging point of view. This misalignment between research evidence and clinical routine results in poor disease free survival rates, mostly due to the lack of non-invasive methods for heterogeneity quantification.

The case study analyzed in this work is composed by a set of $N=333$ lesions belonging to fifty-five patients of Azienda Ospedaliero-Universitaria Pisana with multi-site, multi-lesion, recurrent Prostate Cancer confirmed with a positive PET/CT study. The study was performed in accordance with the Declaration of Helsinki and approved by the local ethics committee (Ethics Committee of the Pisa University Hospital—Pisa 8424/2015). The signature of a specific informed consent and the legal requirements of clinical trials were waived given the observational retrospective study design. During the observational trial, patients showed evidence of biochemical recurrence after first-line treatments, exhibiting metastatic disease. Every patient manifested a different number of tumor lesions $n_i$, according to the spreading burden of the metastatic tumor. Information about age, sex, lesion site, total tumor volume, Gleason Score^19^, Prostate Specific Antigen^20^ and therapy treatment was collected per each patient. Personal information and qualitative tumor data are displayed in Supplementary Tables S1 and S2 online. Additionally, from PET/CT, volumes of interest, i.e. lesions, were segmented by experienced nuclear medicine physicians and texture features were extracted over VOIs according to the radiomic framework, resulting in forty radiomic features ($p=40$). Both the segmentation of lesions and radiomic features extraction were performed using LifeX software^21^, according to the formulas detailed in the software documentation (https://www.lifexsoft.org/).

**Figure 1:**
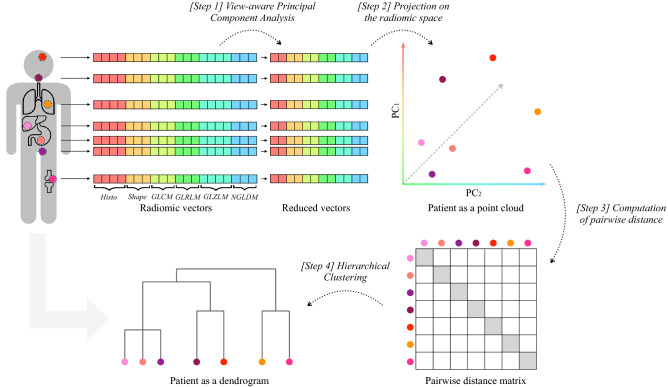
Patient representation pipeline: lesions’ radiomic vectors of each patient are dimensionally reduced according to view-aware Principal Component Analysis. *[Step 1]* Features are grouped according to the six semantic group, or *view*, they are semantically divided into. As to preserve a balanced importance between views, two principal components are kept from the scores of each PCA, leading to different percentages of explained variability. A total of twelve principal components results from the process, which include six orthogonal pairs of linear combinations of original features. *[Step 2]* Accordingly, patients are represented as finite sets of $n_i$ points in ${\mathbb {R}}^{12}$, that is the reduced radiomic space according to view-aware strategy implementation. In the example, $n_i=7$. *[Step 3]* Pairwise (Euclidean) distance is computed among patients’ lesions and *[Step 4]* hierarchical clustering with *average* linkage is applied to distance matrices, resulting in a dendrogram *T* representing each patient.

We fed Prostate Cancer imaging data into the pipeline described in Fig. 1, obtaining a tree-based representation *T* for each of the patients. The pruned edit distance $d_P^\mu$, as defined in the “Methods”, was implemented and leveraged to compute the patient-to-patient distance matrix. Clustering of patients was thus completed according to hierarchical clustering algorithm with the proposed *ad hoc* distance and *ward* linkage. Specifically, *ward* linkage was chosen as it provided better results under a prognostic point of view with respect to other linkages (for a definition of all available linkages see Section 4 of Supplementary Materials online). The number of clusters was selected over the range [2, 5], as a trade off between similarity performance and interpretability. Specifically, the number *k* that presented both a reasonable silhouette coefficient and a high concordance (in terms of mutual information) with therapy response was selected. The resulting classes could then be intended as groups of patients with similar representations in terms of heterogeneous disease, to be characterized according to exogenous clinical variables and risk assessment.

### Clusters characterization

As to profile the clustering, we describe how the stratification procedure captures the differentiation of tumor heterogeneities and provide a clinical/biological interpretation.

**Figure 2:**
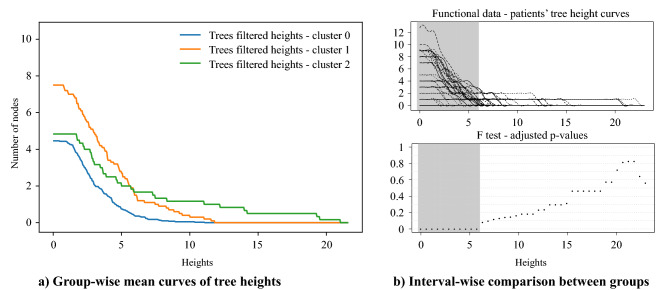
(**a**) Curves displaying the *filtered* heights of the trees’ vertices for the three groups. Operationally, curves were built as follows: for any fixed height (x-axis), for any tree in the selected group, we count the number of nodes whose height value is greater than the fixed one (y-axis). For the step-by-step procedure see Section 3 of Supplementary Materials online. The curves in the plot represent the pointwise within-group means of such counts, and the shaded regions cover an area of 1 standard deviation around the means. The values of such counting process result in a monotonically non-increasing function detecting information about trees’ heterogeneity. In fact, higher values of such function, especially as the height threshold becomes bigger and bigger, correspond to a greater number of heterogeneous lesions in the patients. Patients of group 0 (blue line) are characterized by a very homogeneous disease where trees branches are on average less and very short compared to the other groups; patients of group 1 (orange line) tend to exhibit more lesions than patients belonging to group 0, lesions which are intermediately heterogeneous, as their representation trees display both short branches and longer branches than group 0; patients in group 2 (green line) are associated to very heterogeneous diseases, displaying a similar number of lesions to group 0, but with the associated branches being much longer. A synthetic example of tree per each group is displayed in Fig. 7, elucidating the differences with a graphical support. (**b**) Functional comparison between curves: in order to test the hypothesis that curves belonging to different groups are different, we use the ANOVA procedure proposed in^22^. It outputs an interval-wise adjusted p-value function. Depending on the sort and level $\alpha$ of Type-I error control, significant intervals can be selected. Here, we highlighted in grey the region of significance. Of note, the curves appear different for what homogeneity-heterogeneity balance is concerned; they lose significance as they approach very big height values.

Upon pipeline implementation, hierarchical clustering identified three groups: groups 0, 1 and 2 hosted 39, 10 and 6 patients respectively. In Fig. 2 the curves of the heights of the trees’ vertices over the three groups can be appreciated: branches present different average heights according to the group their dendrograms belong (see Fig. 2). Groups are shown to entail different heterogeneity extent, following an ANOVA functional approach^22,23^.

Beside the group-wise characterization of tree conformation as manifestation of tumor heterogeneity, clinical variables were used as exogenous factors to characterize and interpret the groups. We used appropriate tests according to the variable type, normality of data and sample size. Normality was tested according to the Shapiro test. We thus employed Mann-Whitney non-parametric tests for comparing distributions of continuous (non-normal) variables; parametric t-tests for testing the difference of means in continuous (normal) variables; Levene non-parametric tests for comparing variances of continuous (non-normal) variable; Bartlett parametric tests for continuous (normal) variable ratio of variances; $Chi-squared$ tests for independence of categorical variable. P-values are indicated respectively as $p_{m/d}$ for tests on means/distributions, $p_{var}$ for tests on variance and $p_{ind}$ for tests on independence. Pairwise one-sided comparison between groups rather than multivariate analysis was investigated as to provide a group-wise characterization. As to avoid potential Type II errors due to small sample size, value of $\alpha =0.1$ was considered for significance.

We evaluated the differences between the obtained groups in terms of number of oligo/multi-metastatic patients (as classified with two different clinical cut-offs of 3 and 5 lesions), number of patients with bone disease, total tumor volume and number of tumor lesions. Also, the implementation of combined therapy (such as joint radiotherapy and chemotherapy with respect to only chemotherapy) and response to therapy were evaluated in patients of different groups. Additionally, among clinical prognostic tools, tumor aggressiveness is usually assessed with Gleason Grading System (or Gleason Score)^24^. A Gleason Score (GS) is given to Prostate Cancer based upon its microscopic appearance with respect to cell differentiation. Pathological scores represent the sum of the primary and secondary patterns (each ranging from 1—well differentiated, like normal cells—and 5—poorly differentiated, i.e., abnormal cells) and range from 2 to 10. Higher numbers indicate more aggressive disease, worse prognosis and higher mortality^19^. In particular, patients with Gleason Score exceeding the value of 7 experience extraprostatic extension and biochemical recurrence more frequently than others^25^. Accordingly, clusters were also analyzed in terms of mean Gleason Score and number of patients exceeding GS of 7.

Besides, Prostate Specific Antigen (PSA) has been proposed for screening, assessment of future risk of prostate cancer development, detection of recurrent disease after local therapy and treatment planning of advanced disease. Often employed as criteria in combination of stage and GS, its role in early stage assessments is still debated due to instability of measurements and the presence of confounding factors. However, PSA is still considered a valid tool for prognosis and treatments in advanced stages of metastatic prostate cancer^26^. Moreover, PSA values after cytotoxic regimens has been shown to predict survival. Particularly, the decrease in PSA levels is associated to therapy response in soft tissue lesions and thus could be intended as a proxy of therapy outcome^27^. Accordingly, we recorded PSA levels before the therapy (PSA0), right after the first line of therapy (PSA1) and at the end of the follow up (PSA2). Delta-PSA levels were computed between PSA1-PSA0 and PSA2-PSA0 as proxies of cancer progression. In the following, they will be referred as PSA, $\Delta PSA_{1,0}$ and $\Delta PSA_{2,0}$.

**Table 1:** Significance in terms of *p* values of the statistical tests between cluster 0 and cluster 1, cluster 0 and cluster 2, cluster 1 and cluster 2 in the proposed pipeline: non-parametric/parametric tests on difference of averages and variances were performed for (non-normal/normal) numerical variables while tests on category independence were performed for categorical variables.

Variable	Test on	0 versus 1	0 versus 2	1 versus 2
		(*p* values)	(*p* values)	(*p* values)
Gleason score	Mean	0.2967	**0.0419**	**0.0601**
	Variance	0.8368	0.5433	0.7093
Gleason category	Independence	0.5129	0.5056	0.3077
Oligo or multi ($>3$)	Independence	**0.0601**	0.9260	0.1729
Oligo or multi ($>5$)	Independence	**0.0848**	0.6868	0.3339
$3<$ Lesions $\le 5$	Independence	0.1969	0.9022	0.3950
N lesions	Mean	**0.0081**	0.4162	**0.0722**
	Variance	0.3871	0.4357	0.1469
Skeleton	Independence	**0.0769**	0.9622	0.1729
Total volume (ml)	Mean	**0.0002**	0.4917	**0.0306**
	Variance	**0.0000**	**0.0047**	0.2009
PSA	Mean	**0.0116**	0.3787	0.3089
	Variance	**0.0013**	0.4714	0.1845
$\Delta PSA_{1,0}$	Mean	**0.0019**	0.3477	0.1810
	Variance	**0.0003**	0.4533	**0.0995**
$\Delta PSA_{2,0}$	Mean	0.3689	**0.0591**	0.1855
	Variance	**0.0066**	0.2159	**0.1085**
Ongoing therapy	Independence	**0.0601**	0.5875	0.3339
Combined therapy	Independence	**0.0517**	0.6091	**0.0863**
Therapy response	Independence	0.6856	0.127	0.2907

Significant values are in [bold].

**Figure 3:**
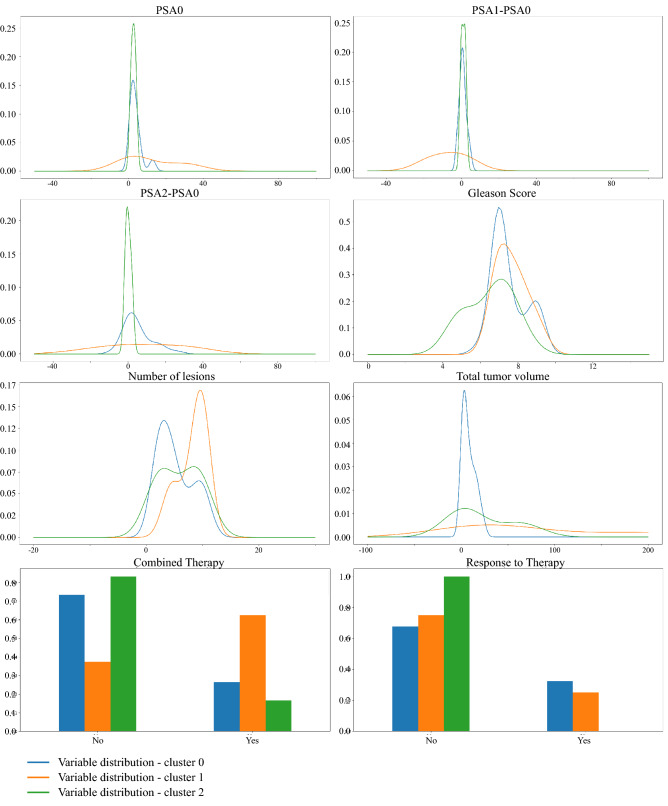
Results of clustering characterization: the first three rows draw the distributions of the numerical clinical variables in the three groups, namely the PSA values, the $\Delta PSA_{1,0}$, the $\Delta PSA_{2,0}$, the number of lesions, Gleason Scores and the total tumor volume; the last row shows the proportions of the categorical clinical variables in the three groups, that are the combination of therapy and the response to treatment. For the proportion of skeleton disease and of the oligo/multi-metastatic status as devised by the two clinical cut-offs (3 and 5 lesions) see Section 8 of Supplementary Materials online.

Table 1 and Fig. 3 elucidate the results. The profile of the blue and green groups are very similar for what PSA ($p_{m/d}=0.3787$, $p_{var}=0.4714$) and $\Delta PSA_{1,0}$ ($p_{m/d}=0.3477$, $p_{var}=0.4533$) are concerned, with a very limited range of values concentrated around zero. Different trends are exhibited by the blue and green curves of the $\Delta PSA_{2,0}$ ($p_{m/d}=0.0591$), where the difference could support the hypothesis of different cancer evolution starting from similar baseline assessments. Yet, they present similar variance ($p_{var}=0.2159$). The orange group, on the other hand, presents wider ranges and higher intra-group heterogeneity. In particular, orange PSA is significantly higher than the blue group with a much more spread distribution ($p_{m/d}=0.0116$; $p_{var}=0.0013$) yet no statistical difference with the green groups is confirmed ($p_{m/d}=0.3089$; $p_{var}=0.1845$); orange $\Delta PSA_{1,0}$ is significantly lower than the blue group ($p_{m/d}=0.0019$) but not than the green one ($p_{m/d} = 0.1810$), however its distribution appears more spread and inhomogeneous, covering both the negative and the positive axis, in both cases ($p_{var}=0.0003$; $p_{var}= 0.0995$). The $\Delta PSA_{2,0}$ of the orange group does not vary from the one of the blue group ($p_{m/d}=0.3689$). However, it shows a higher variance than the other, suggesting a heterogeneous long-term tumor prognosis ($p_{var}=0.0066$). Also, the orange group and the green group do not differ significantly in their average ($p_{m/d}=0.1855$) but their variances reveal a mild divergence in terms of distribution kurtosis ($p_{var}=0.1085$).

Regarding the number of lesions, the orange group displays a higher number of metastases than the blue one ($p_{m/d}=0.0081$). The green group exhibits a behavior very similar to the blue group ($p_{m/d}=0.4162$), diverging from the orange group with respect to which it presents fewer lesions ($p_{m/d}=0.0722$). Moreover, total volume of the tumor is related to the number of lesions. In fact, the blue group displays a reduced spreading of the tumor over the body with respect to the orange group ($p_{m/d}=0.0002$) but not to the green group ($p_{m/d}=0.4917$). The orange and the green groups also exhibit a statistical difference in terms of tumor volume ($p_{m/d}=0.0306$). Of note, despite the number of metastases in the blue and green groups are very similar, it should be noticed that their tumor spreading appears shifted in the figure, entailing unrelated tumor burden information. Similarly, the orange group, while presenting a greater number of lesions, shows an extension of the tumor visually analogous to the green group. Such discrepancy is imputable to the difference of variances the distributions display.

From these consideration, it appears clear how the green group shows phenotypic similarities and dissimilarities with respect to both blue group and orange group, presenting an in-between behavior. However, the detach of green patients from the rest of the population is mostly driven by the different distribution of GS levels. In fact, the blue and orange groups do not show peculiar differences ($p_{m/d}=0.2967$), although both differ from the green group, compared to which they have a higher GS ($p_{m/d}=0.0419$; $p_{m/d}=0.0601$). As it will be further discussed in discussion, prognostic power of GS values should be taken with the grain of salt due to their qualitative and aggregated nature.

As for the clinical assessment of patients, the blue and green groups present similar to each other yet opposite characterizations with respect to the orange group. They display a lower percentage of patient with bone disease ($p_{ind}=0.0769$; $p_{ind}=0.1729$), therefore fewer people who have undergone an invasive combination of therapies ($p_{ind}=0.0517$; $p_{ind}=0.0863$). Moreover, although the results on the response to therapy are not significant due to the limited data available, they reveal a certain trend. In fact, both blue and green groups of patients are administered a milder therapy with respect to orange group. On one hand, such treatment results effective for the blue group, which shows the highest percentage of responders; while, on the other hand, this is not the case for the green group, which manifests the highest percentage of non-responders. Group 2 thus exhibit a clinical characterization comparable to group 0, whereas tree conformation analysis and prognostic assessment, i.e., response to therapy, agree in granting it a higher score of risk. Finally, the orange group presents the highest number of multi-metastatic patients, followed by the blue group and finally the green group, which hosts mostly oligo-metastatic patients.

**Figure 4:**
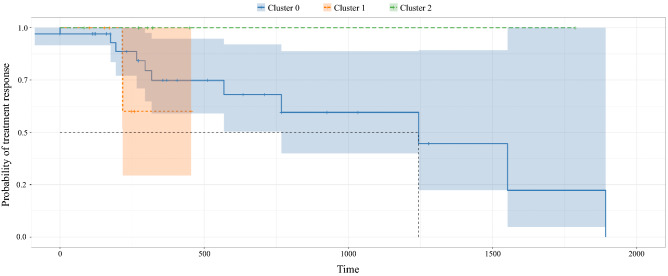
Group-wise Kaplan Meier curves of time to therapy response: it visually shows the probability of the response to treatment in a certain time interval. The blue line, the orange line and the green line correspond to group 0, 1 and 2 arising from clustering performed on patients’ dendrograms. Groups have a different time to response. In particular, green group does not respond to therapy along the study period. Orange group shows indeterminate results due to the lack of and heterogeneity of clinical data. Blue group gradually responds throughout the study period.

From Fig. 4, some extent of stratification is appreciable, although the groups’ survival curves separation is not neat and statistically significant ($p = 0.12$). All patients of group 0 gradually respond since they feature mild disease, both from a structural, i.e., tree conformation, and clinical point of view. The green group host patients who the clinic would treat as not severe (in terms of number of lesions, GS and PSA baseline information), but our radiomics investigation has put in an at risk group, to be properly monitored, in terms of tree structure and tumor extension. In line with the results of our policy, these patients do not respond to therapy during the study period. Finally, the orange group carries severe patients from both a structural and a clinical point of view.

Since unsupervised approaches are thoroughly dataset dependent, hierarchical clustering grouped in the same clusters very heterogeneous patients, due to the limited data available. In fact, clinical variable variance of orange patients was consistently larger than other groups—despite not being the largest cluster. Interestingly, we fit a DBSCAN (Density Based Spatial Clustering of Applications with Noise) algorithm^28^ on the pruned-edit distance matrix which lead to the same clustering policy of patients. In this setting, while blue and green groups were confirmed to be clusters with similar density, the orange group was classified as noise, i.e., observations that display inconsistent density characterization. Accordingly, a couple of patients responded to therapy while the majority did not respond and entered more invasive treatments. For these reasons, the orange survival curve is hardly interpretable and is left out the discussion. For sure, the high variability of this group testifies that a larger testing cohort would allow to identify further separations within this group, leading to clearer prognostic results.

### Comparison with state-of-the-art methods

The established radiomics frameworks contemplate the extraction of texture features from a single lesion, often located on the prostate where the bigger lesion or the primary tumor are found. Such features are usually fed into a classification or stratification model as to predict cancer diagnosis, staging and prognosis.

As a comparison with the state of the art, we investigated the stratification resulting from the analysis of the biggest lesions’ textural description. We selected the bigger lesion of each patient, we reduced the texture vector dimensionality according to view-aware PCA dimensionality reduction procedures and we performed hierarchical clustering on the patient-to-patient Euclidean distance matrix with *ward* linkage. The clustering procedure lead to the stratification of patients into two groups, namely group 0 and group 1. It is worth noting that this clustering approach—based only on the bigger lesion and/or primary tumor—share some extent of the stratification underpinnings of the tree-based clustering. For the sake of clarity, we refer to one-lesion clustering as *tumor clustering* and to tree-based clustering as *heterogeneity clustering*. In particular, tumor clustering resulted to have a mild concordance with heterogeneity clustering (Rand Index $=0.43$^29^). Coherently, the tumor-based stratification leads to clinical significance. Tumor clustering pipeline discriminated between patients with different GS ($p_{m/d}=0.0259$), number of lesions ($p_{m/d} = 0.0001$), oligo/multi-metastatic disease proportions ($p_{ind}=0.0191$), PSA ($p_{m/d}=0.0339$), ongoing therapy ($p_{ind}=0.0847$) and total volume ($p_{m/d} < 0.0001$). However, $\Delta PSA_{1,0}$ ($p_{m/d}=0.2942$), $\Delta PSA_{2,0}$ ($p_{m/d}=0.2920$), proportion of patients exhibiting bone disease ($p_{m/d}=0.5220$), combination of therapy ($p_{ind}=0.3698$) and response to therapy ($p_{ind}=0.2170$) did not result significant in tumor clustering pipeline. These findings were somehow expected. In fact, therapeutic guidelines are mainly taken on the basis of the characterization of the primary tumor. Accordingly, these results confirm the role of the primary tumor in acting as a driver for tumor heterogeneity and enforce radiomics role in the clinical treatment planning. Nevertheless, despite the coherence with qualitative clinical investigation, tumor-based stratification does not translate into a risk assessment and prediction. In fact, the Kaplan Meier curve, describing the probability of response to treatment of the two groups, appear almost superimposed ($p = 0.85$) and do not reveal any prognostic mechanism of the clustering.

As a step forward from one-lesion strategy, radiomics literature suggests to average radiomic descriptions of peer lesions belonging to a patient, as to obtain one single vector. Such vector-based representation plays for the mean imaging phenotype of all lesions expressed by a patient, taking into account the variability of the imaging profiles. Such method provide an information-complexity trade-off between one-lesion strategy and the tree-based patient representation we propose. Under these considerations, we performed patient-wise weighting of lesions’ vectors, implemented the view-aware PCA dimensionality reduction methods and computed vector-based representation of each patient. The pipeline grouped all the patients in one cluster, although one patient with higher PSA was clustered separately from the rest of the cohort population as to meet hyperparameter criteria (e.g. minimum number of clusters at least equal to 2). Clear stratification was indeed not achieved in this setting, however a particularly bad-prognosis patient detached from the main group. From these findings, it follows that vector-based representation model did not lead to clear and solid results in our dataset, suggesting the non robustness of the lesions’ weighting procedures.

## Discussion

Current radiomic framework presents some limitations, including the inter-operator variability in imaging acquisition settings, the relatively small sample sizes bounding the performance of supervised approaches, the lack of standardization, the high dimensionality and the collinearity of radiomics variables as well as the absence of a clinical interpretation for features^30^. For these reasons, intra-patient tumor heterogeneity quantification has long been attempted with poorer results, hampering its embedding into daily practice. In this work, we propose a patient representation for agnostic multi-lesion cancer description, able to overcome intrisinc limitations of radiomics. The method exploits the texture analysis of lesions’ imaging according to the radiomic workflow, overcoming features redundancy with PCA-based dimensionality reduction strategies. The proposed dendrogram representation results *agnostic* with respect to acquisition settings and operator variability as it is built upon statistical relationship within peer lesions’ descriptions. Moreover, the small sample size issue is tackled by the employment of unsupervised methods. As to leverage the complex representation for stratification purposes, a suitable distance between dendrograms was required. Indeed, the pruned tree edit distance was specifically designed for heterogeneity-based hierarchical dendrograms and was the keystone to deliver a stratification policy based on agnostic disease conformations.

For what dimensionality reduction is concerned, view-aware PCA was hereby proposed as a scalable yet minor novelty. PCA is a well known, established, interpretable technique, often producing good results. With the rationale of improving the scalability of the approach, we introduced the view-aware PCA, such that the dimensionality reduction step could be performed in parallel in smaller and semantically-similar euclidean spaces. Nevertheless, further studies could investigate the sensitivity of the representation model and the performance of the clustering policy when employing feature transformation methods that capture non-linear dependencies of across- and within-view features^31^.

Compared to state-of-the-art disease representation, our pipeline shaped an exhaustive representation of intra-patient heterogeneity and devised an informed patient stratification. In fact, it led to a more complex yet low-processed modelling of cancer disease, underlining interactions and relationships between lesions of individuals from which to infer prognostic knowledge. Clearly, one-lesion strategy did not provide a quantification of lesions’ diverse phenotypes within a patient, as it only relies on the primary tumor. Nevertheless, tumor clustering led to a coherent stratification with respect to the current clinical biomarkers, i.e., PSA, GS and oligo/multi-metastatic status. However, such clinically-informed stratification did not reach a significance in terms of prognostic power, bringing out the limitation of current clinical and radiomic-based biomarkers for treatment and prognosis. Interestingly, the proposed representation brought out a comprehensive way to capture tumor biology and heterogeneity, revealing a deeper appreciation of the disease than a single lesion or the primary tumor alone. On the other hand, the vector-based representation was confirmed insufficient to properly embed the patient’s complexity of information. In fact, mean radiomic profile seemed not to properly capture intra-tumor variability while it overlooked the primary tumor information entailing clinical information. In both cases—when only the primary tumor was considered and when the mean radiomic profile of lesions was computed - state of the art methods failed in perspectively stratifying patients.

Beside descriptive and prognostic purposes, the proposed tree-based representation and stratification of tumor heterogeneity permitted an exhaustive comparison between the role played by the primary lesion and its involvement into phenotypic selection mechanism. This is worth to be drawn and further investigated from a tumor heterogeneity and prognostic point of view. In fact, tumor clustering showed a latent agreement with heterogeneity clustering, suggesting the reliability of the current clinical practice in assessing intra-tumor characterization from primary lesions. Accordingly, primary tumor information seemed to be more informative than intra-patient mean lesions’ profiles. If used in combination with dissemination indexes—such as number of metastases, dispersion of intra-patient lesions’ radiomic profiles and number of involved organs -, primary tumor characterization could provide enough information to support therapeutic decisions when an exhaustive assessment of tumor metastases results too expensive.

Of note, heterogeneity clustering highlighted milder significance for what GS biomarkers is concerned with respect to tumor clustering. Pertinently, although GS is a solid clinical prognostic factor driving therapy planning, it represents the histo-pathological analysis for characterizing primary and secondary tumor biology at molecular level. Accordingly, the aggregated value, that is the sum of primary differentiation pattern and secondary differentiation pattern, do not entail heterogeneity information. For instance, studies using surrogate PCa end points have suggested that outcomes for GS 7 cancers vary according to the predominance of pattern 4. PCa mortality, biochemical progression and development of metastases differ for 3 + 4 and 4 + 3 tumors^32^. This means that, according to tree-based representation, patients tagged with a GS 7 may still be clustered in different prognostic groups and alter the tests on averages. For these reasons, GS should not be considered as a solid ground truth for a perspective model, rather it conveys only a association between radiomic-based heterogeneity assessment and its biological counterpart, that is tumor microscopic appearance. On the other hand, PSA and $\Delta PSA$ values significantly supported the predictive power of imaging-based representation in terms of cancer progression and disease free survival. Consistently, a decrease in PSA levels after treatment regimens was associated to therapy response. In this sense, exhaustive lesions’ texture assessment and imaging-based heterogeneity quantification devised cancer subtypes that correlated with prognosis beyond clinical surrogates, eventually supporting treatment planning.

Basing on our and literature findings, the systematic digital tissue collection and its analysis should be enforced in the translational research of tumor disease and in the developing of targeted therapies. The debate around the therapeutic exploitation of imaging biomarkers for intra-tumor heterogeneity is nowadays on the cutting edge of medicine literature and it interlaces with other science field such as mathematics and geometry. This dynamic interplay between disciplines may provide a propitious route to ultimately attempt to limit tumor progression and treatment resistance. Stemming from this work, future research could consider longitudinal evolution of heterogeneity-based representation objects and, accordingly, investigate the course of the disease over time in a non invasive way.

## Methods

In this section we outline the steps involved in the proposed methodological pipeline. In particular, methods for radiomics-based representation of patients’ heterogeneity and its stratification are discussed. We present the challenges of analyzing a general radiomic dataset proposing an insightful dimensionality reduction approach (M1). Representation strategy is then deduced and described (M2). We then introduce an existing edit distance for comparing tree objects, on which we build the proposed metrics. It follows the derivation of an *ad hoc* metric (M3) for capturing intra-tumor heterogeneity variability and computing the similarity matrix between patients on which to perform the stratification according to hierarchical clustering.

### M1: dimensionality reduction

As previously introduced, radiomic features are regarded as a high dimensional vector embedding of the VOI, providing a non-invasive assessment of tumor appearance from routinely acquired imaging studies. Several softwares, e.g. LifeX software, allow to extract several texture indexes from VOIs, according to the formulas provided by the software documentation (https://www.lifexsoft.org/). Considerable efforts have been devoted to link biological meaning with texture descriptors. So far, little evidence of tight correlation between the two has been found, preventing from univocally define tumor inherent heterogeneity of lesions. However, different textural features have been proposed and reviewed by Castellano et al.^33^ as measures of tumor-specific intra-lesion heterogeneity. Indeed, radiomics analysis is widely assumed to entail all the information needed for a definition of lesion heterogeneity^34,35^.

When managing a radiomic dataset, several challenges come across, above all high dimensionality and collinearity between features. Thus, prior to pairwise distance computation, lesions’ radiomic vectors need to be properly reduced as to selectively bring out relevant information.

According to Nioche et al.^21^, radiomic features divide into six semantic groups of different methodological levels of texture analysis. *First order statistics* are the statistical moments of the grey level distribution extracted from the VOI under analysis. *Shape features* describe morphological characteristics of the tumor. The *Grey Level Co-occurrence matrix* (GLCM) describes the co-occurrence of pairs of grey values in the VOI at a given distance $\delta$ (offset), usually set to 1, towards thirteen different directions. The *Grey Level Run Length matrix* (GLRLM) describes the length of homogeneous *runs* for each grey level, averaged across thirteen directions. Similarly, the *Grey Level Zone Length matrix* (GLZLM) provides information on the size of homogeneous *zones* for each grey level, averaged across three dimensions. Finally, the *Neighbour Grey Level Difference matrix* (NGLDM) corresponds to the difference of grey levels between one voxel and its twenty-six neighbors in three dimensions. From each of these groups, several indices are extracted, exhibiting a multi-view intrinsic structure that induces intra- and inter-group correlation patterns. Accordingly, such vectors disclose high collinearity between their elements that needs to be properly managed. To overcome this, we leverage the very basic idea of multi-view learning and dimensionality reduction approaches: the view-wise linear combination of features^36^. We propose to separately apply the PCA to each of the radiomic groups, as to exploit the multi-view nature of the radiomic vectors and to reduce the computational cost of the dimensionality reduction step. In this way, we may keep the information carried by each group well discerned, as it is methodologically extracted in different ways. A more interpretable  and scalable dimensionality reduction comes from the process.

Upon pre-processing, namely missing values imputation and Z-transform normalization of radiomic variables, we thus perform this novel dimensionality reduction, namely *view-aware PCA*.

As depicted in Fig. 1, features are grouped according to the six semantic group—*view*—as described above. Within each group, PCA is performed and two principal components are retained from the scores of each PCA, resulting in different percentages of explained variability. The process yields a total of twelve principal components, including six orthogonal pairs of linear combinations of original features. It follows that each lesion is described by a twelve-dimensional vector entailing view-wise texture information.

Further, we build the patient representation upon the such reduced radiomic vectors of peer lesions.

### M2: tree-based patient representation

To exhaustively represent patients’ disease in terms of tumor heterogeneity, relationships between lesions needs to be learnt from data. Distance between texture descriptors could be an appropriate surrogate. Specifically, radiomic variables of a lesion—possibly after dimensionality reduction as in M1—define a lesion-specific point in an Euclidean space. All lesions belonging to the same patient form a point cloud in ${\mathbb {R}}^p$, with a number of points $n_i$ equal to the number of patient’s tumor lesions and *p* being the number of radiomic variables.

Although some frameworks are available to compare point clouds via discrete transport^37,38^, interpretability is often limited by the high dimensionality of the embedding space. Also, model based approaches, which capture the variability of cloud-generating processes by means of interpretable parameters, require a high number of observations in each point cloud to produce reliable estimations^39^.

**Figure 5:**
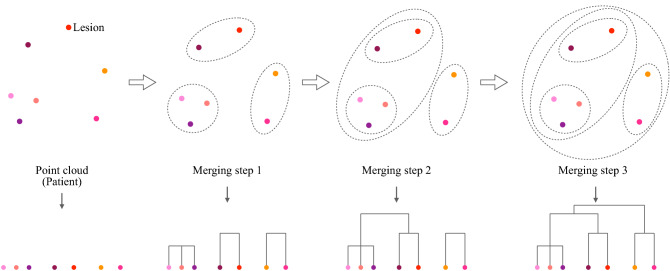
Tree-based patient representation via agglomerative hierarchical clustering: from the bottom up to the root, leaves get agglomerated and merged into bigger and bigger clusters, to finally converge in a single set. As a consequence, tree branches reflect pairwise similarity between lesions and the tree structure surrogates the overall dispersion among peer lesions. In the final dendrogram representation, leaves are the lesions of the patient and edges illustrate the similarity-connection between them. Leaves that are close to each other are intended by construction to be similar and exhibit a comparable radiomic profile (homogeneous) while distant leaves can be thought as lesions expressing different imaging phenotypes (heterogeneous). In this sense, dendrogram structure entails the heterogeneity quantification within the tumor, which needs to be exploited for heterogeneity-based stratification of patients. For mathematical formulation see Section 4 of Supplementary Materials online.

A more insightful approach would be to transform the point cloud into a proper summary, i.e., a representation, equally informative and easily readable. Pertinently, hierarchical clustering dendrograms have been extensively studied in the last decades as they unveil the intrinsic relationship among points of a point cloud (for a review on hierarchical clustering dendrograms see^40^). In our setting, the rationale behind hierarchical clustering stems from the need to quantify to which extent lesions, i.e., their radiomic vectors, are similar within patients and how they get agglomerated, hierarchically, one to each other. A dendrogram is obtained in such a way that lesions are linked in terms of relationship, based on similarities in their imaging characteristics. Figure 5 graphically describes the process while Section 4 of Supplementary Materials online formalizes the mathematical steps involved. Dendrograms’ structure reflects the homogeneity between points of the point cloud. For instance, Fig. 7 presents three dendrograms: the blue one describes a condensed point cloud, the green one presents a scattered point cloud while the orange tree denotes a hybrid situation.

To build hierarchical clustering dendrograms, a similarity measure is needed together with an agglomerative criterion—also known as *linkage*—that best suit the structure of the data and the aim of the analysis. In our setting, an appropriate similarity measure is the Euclidean distance between lesions’ radiomic vectors, as suggested by Cavinato et al.^13^. Additionally, *average* linkage is employed as it is known to be less sensitive to outliers, producing a more robust representation^41^.

### M3: a novel heterogeneity-based distance

After having obtained patient representation, we proceed to defining a distance between dendrograms, which can properly reflect the affinity between patients in terms of tree conformations as manifestation of intra-tumor heterogeneity. A suitable metric should meet some requirements in order to produce effective results: (1) the comparison between dendrograms should reflect the properties of the point cloud they stem from: if two point clouds are close in terms of sparsity and conformation, we require the associated dendrograms to be close as well. In other words, any metric between dendrograms must hold some *continuity* properties with respect to the original point clouds comparison; (2) the metrics should weight differently the homogeneous part of the tree structures and the heterogeneous ones. This means that distance has to be evaluated as a trade-off between the extents of homogeneity and heterogeneity exhibited by the lesions of different patients.

#### Edit distance

Dendrograms are *unlabelled* object which, in our context, may have a different number of leaves and do not hold any a-priori correspondence between the leaves in different objects.

The literature dealing with the comparison of dendrograms is reviewed Section 2 of Supplementary Materials online, where we detail the limitations that prevent us from employing existing distances in our context. Recently, Pegoraro et al.^42–44^ proposed a novel distance for merge trees. Following the authors, we call this metric *edit distance* for merge trees and indicate it with $d_E$. The metric $d_E$ is defined for weighted, rooted, unlabelled trees and extends to merge trees via truncation process (see Section 4 of Supplementary Materials online and ^43^). As most of the metrics for unlabelled trees, its computational complexity has been shown to scale poorly with the number of leaves in the trees. However, it is particularly efficient for small-scale trees with respect to other metrics. In our setting, trees present a number of leaves less or equal to the number of tumor lesions in a patient, that is a few dozens at most. Thus, we can run the comptuation of $d_E$ on general purpose machines, like personal computers. Unlike other metrics, continuity properties are easily proven. Moreover, $d_E$ is interpretable, easy to understand and to communicate.

**Figure 6:**
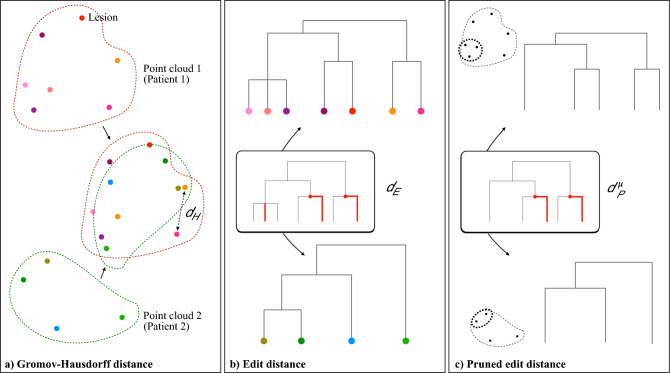
Continuity among metrics. (**a**) Gromov-Hausdorff distance between two point clouds: the point clouds get overlapped and $d_H$ is defined as the maximum distance between the two maximally distant points; Gromov-Hausdorff-closeness reflects the similarity in the spreading of points of two point clouds throughout the space. Specifically in the radiomic space, such spreading entails the quantification of inter-patient heterogeneity. This means that Gromov-Hausdorff-close point clouds, i.e., patients’ sets of lesions, have similar intra-patient heterogeneity characterization and thus should be regarded as similar by the metric we employ for dendrograms; (**b**) Tree edit distance $d_E$ between hierarchical clustering dendrograms: the distance is given by the sum of the costs of the minimum number of modifications needed for transforming a tree into the other. Modifications include positive/negative shrinking, deletion/insertion and ghosting/splitting. The *shrinking* edit multiplies the weight value of an edge with a positive factor, which can either lengthen (positive shrinking) of shorten (negative shrinking) the original edge weight. The cost of shrinking an edge is equal to the absolute value of the difference between the initial and the final weights. *Deleting* or *inserting* an edge $(v_1,v_2)$ removes or introduces a branch at a given height, altering the children-father structure of the tree. For any deletion/insertion, the cost is equal to the weight of the edge deleted/inserted. Finally, the *ghosting* edit eliminates a vertex $v$ that connects only two adjacent edges (order 2 vertex) such as one new edge results from the sum of the two former edges. The opposite edit is the *splitting*. Ghosting and splitting have no cost, therefore order 2 vertices are *de facto* irrelevant when computing the cost of an edit path; (**c**) Pruned tree edit distance between pruned dendrograms: pruning removes leaves with weights $\le \varepsilon$, eventually aggregating homogeneous phenotypes. The operator $P_\varepsilon$ thus gradually discards intra-patient homogeneity, disclosing only the heterogeneous—independent—tumor phenotypes. Of note, $d_P^{\mu }$ is different from $d_E$ since the pruning modulates the effect of cardinality on the distance computation by removing redundant edges of the tree and compressing tree dimensionality.

As depicted in Fig. 6b), one tree *T* can be modified and transformed into a different tree $T'$ by performing different sets of allowed modifications, each coming with its own cost (for details see Pegoraro et al.^42,43^). The set of consequent edit operations which comes at the minimum cost is named the *optimal edit path* and represents the core of the edit distance between the two trees. The distance $d_E$ is thus the total cost of the optimal edit path and is defined as:

1 $$\begin{aligned} d_E(T,T')=\inf _{\gamma \in \Gamma (T,T')} cost(\gamma ) \end{aligned}$$

where $\Gamma (T,T')$ indicates all the possible edit paths which start in *T* and ends in $T'$. The algorithm for $d_E$ computation is exhaustively detailed in^42^. Through combinatorial objects called *mappings*, it is shown that $d_E$ is a metric in the space of merge trees and that it can be computed with a Linear Integer Programming approach^42^.

Upon these premises, we proceed to verify the two aforementioned conditions. Specifically, (1) we prove the continuity property of $d_E$ and (2) propose a modification of $d_E$ as to meet the homogeneity-heterogeneity requirement.

#### Continuity property of *d*_E_

As previously stated, a continuity result with respect to the original point clouds comparison would guarantee interpretability properties for the distance between dendrograms: under certain hypotheses, if two clouds are pointwise close, also their merge trees should be close with respect to $d_E$. In Fig. 6a), we introduce the Gromov-Hausdorff metric between point clouds (for formal definition see Section 5 of Supplementary Materials online). It can be interpreted as a measure of the pointwise proximity between two point clouds and provide a comparison between the heterogeneity of two patients’ diseases. In Section 5 of Supplementary Materials online, we prove that Gromov-Hausdorff-closeness for point clouds implies Edit-closeness for the associated dendrogram objects, i.e., multi-lesion patients representation.

#### Homogeneity-heterogeneity trade-off

In the edit distance $d_E$, the distance values are strongly dependent on the clouds cardinalities, meaning that pairs of point clouds with higher cardinalities tend to be farther apart from pairs of point clouds with smaller cardinalities. At first sight, such assumption sounds reasonable for stratification purposes. In fact, patients with multiple lesions are known to exhibit a more severe disease than patients with fewer lesions, as the spreading of the tumor entails prognostic power. Still, the mere counting of lesions lacks of robustness in perspective studies and, in this context, may overshadow the variability between hierarchical dendrograms induced by intra-patient heterogeneity. For this reason, we propose a modification of the metric $d_E$ as to mitigate cardinality issue.

#### Pruned edit distance

The kind of variability we are interested in is the one induced by patient-wise heterogeneity between lesions. By construction of the dendrogram representation, two lesions of a patient are heterogeneous—in terms of radiomic/imaging description—according to the length of the dendrogram branches connecting them. The longer the branches, the higher the inter-lesions heterogeneity and, viceversa, the shorter the branches the more homogeneous the patient’s disease phenotypes. Accordingly, we may want to modulate the extent to which we consider edit costs according to branch length. In particular, we may want to induce edits applied on small edges to contribute less to the final distance than bigger edges, which we deem more relevant for stratification purposes.

We introduce the pruning operator $P_\varepsilon$ as regularization strategy, which deletes leaves associated with edges whose weights are so small that one may want to neglect them in the analysis of heterogeneity. Given a threshold $\varepsilon$, we consider for deletion all leaves whose father-child edge has weight $\le \varepsilon$. However, when two or more of candidate leaves share the same father, i.e. they are *siblings*, we delete all the leaves but the one with the bigger weight. Moreover, if the weights of the siblings are equal, as it is often the case in clustering dendrograms, we randomly choose to keep one of them, delete the other(s) and, eventually, *ghost* their father (see Fig. 6 for meaning of ghosting). This pruning operation is recursively iterated until no leaves with small edges can be found. To note, removing only one leaf in case of two small-weight siblings is equivalent to considering the two leaves as clustered together from the “beginning” in the hierarchical clustering procedure. Accordingly, siblings leaves (lesions) entail phenotype expressions so similar to be considered as one single imaging phenotype. In this way, the pruned tree displays the number of *different* phenotypes coexisting in the patient instead of the mere number of lesions. Figure 6c) displays the edits needed for transforming a pruned tree into another, whose costs determine the pruned edit distance.

**Figure 7:**
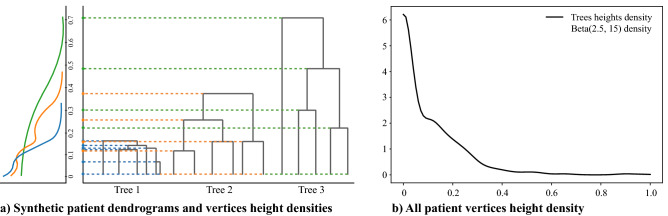
Choice of $\mu$: (**a**) costruction of qualitative densities of the vertices heights in three example dendrograms: the velocity with which leaves get merged in a dendrogram, i.e., edges length variability, reflects the heterogeneity characterization of lesions. Per every dendrogram, branches heights (rescaled on [0, 1] dividing by the highest value) are annoted on the left and their associated density is inspected. The vertices heights of a patient exhibiting homogeneous lesions concentrates in a small real interval [0, *a*]—with $a>0$ (blue tree); the vertices heights of a patient exhibiting heterogeneous lesions spread in a range of values far from zero [*a*, *b*], with $a,b>0$ (green tree); a patient showing groups of homogeneous lesions, the one heterogeneous to the others, is associated to a dendrogram with an explicit clustering structure with clusters with multiple close leaves (orange tree). The vertices heights distribution displays two components, reflecting both the homogeneity of similar lesions—with values close to 0—and the heterogeneity of dissimilar clusters—with values far from 0; b) $\mu$ provides the coefficients with which to weight the different pruning cutoffs $\varepsilon$, to neglect the homogeneity within clusters of similar lesions’ phenotypes and bring out the informative heterogeneity between different phenotypes. To efficient the computation, a parametric shape of $\mu$ is used and empirical heights distributions of all patients (black line) is exploited to model the distribution. In the population heights distribution, we discern both homogeneous and heterogeneous phenotypes. The two components are demarked with a saddle point on 0.15. Accordingly, low weights of $\mu$ should be associated to $\varepsilon \ll 0.15$ and $\varepsilon \gg 0.15$ and high weights to $\varepsilon \simeq 0.15$. In fact, low $\varepsilon$ values entail pure homogeneity information while high $\varepsilon$ values would lead to discarding useful heterogeneity information. We thus infer to model $\mu$ as an asymmetric bell-shaped density function with one peak centered in the saddle point of the heights distribution. The Beta family of distributions, supported in [0, 1], well meets the requirements; it simplifies both the numeric integration procedure and the results’ interpretation. The Beta-shaped $\mu$ is centered on 0.15 (grey line), properly tuning $\alpha$ and $\beta$ shape parameters ($\alpha =2.5,\;\beta =15$).

Operationally speaking, the “correct” value of $\varepsilon$ is a-priori unknown and needs to be tuned with a complexity-information trade-off. To enhance the robustness of this parameter choice, we take the weighted average of the distances between two trees pruned with all the possible values of $\varepsilon$. Accordingly, the definition of *pruned edit distance* for general merge trees develops as follows. Given two merge trees *T* and $T'$, the pruned edit distance is:

2 $$\begin{aligned} d^\mu _P(T,T'):=\int _{{\mathbb {R}}} d_E(P_\varepsilon (T),P_\varepsilon (T'))d\mu (\varepsilon )= {\mathbb {E}}_{\varepsilon \sim \mu }[ d_E(P_\varepsilon (T),P_\varepsilon (T')] \end{aligned}$$

where $\mu$ is a finite measure on ${\mathbb {R}}$ which provides the weighting strategy across different values of $\varepsilon$ in order to compute a weighted average among trees distances. The higher the mass $\mu$ associated to an interval [*a*, *b*], the bigger the contribution to the final result of the tree distance according to $\varepsilon \in [a,b]$. In other words, the measure $\mu$ allows to control the contribution to the final distance of branches with weight below $\varepsilon$, which are indeed homogeneous enough to be removed. Figure 7 elucidates the choice of $\mu$ tuned on case study data. Note that if we have a sequence of weakly converging probability measures $\mu _n \rightharpoonup \mu$, then $d^{\mu _n}_P(T,T') \rightarrow d^{\mu }_P(T,T')$. This implies that the proposed distance is robust with respect to the choice of $\mu$: similar measures $\mu$ (in the sense of weak convergence) would give similar distances.

To assess the different behaviours between $d_E$ and $d^\mu _P$ and the extent to which $d_P^\mu$ is suitable for our purposes, in Section 7 of Supplementary Material online we present a detailed simulation study. Moreover, we can prove that, under general conditions on $\mu$, $d_P^\mu$ is still a metric (for proof see Section 6 of Supplementary Materials online).

## Supplementary Information

Supplementary Information.

## Data Availability

The data that support the findings of this study are available from Azienda Ospedaliero-Universitaria Pisana but restrictions apply to the availability of these data, which were used under license for the current study, and so are not publicly available. Data are however available from the authors upon reasonable request and with permission of Azienda Ospedaliero-Universitaria Pisana. The code implemented during the current study together with simulation data will be available upon acceptance on GitHub at this link https://github.com/pego91/pruned-edit-distance.
