# Author Correction: Imaging-based representation and stratification of intra-tumor heterogeneity via tree-edit distance

**DOI:** 10.1038/s41598-022-27001-4

**Published:** 2022-12-29

**Authors:** Lara Cavinato, Matteo Pegoraro, Alessandra Ragni, Martina Sollini, Paola Anna Erba, Francesca Ieva

**Affiliations:** 1 MOX Lab, Department of Mathematics, Politecnico di Milano, 20133 Milan, Italy; 2 Department of Biomedical Sciences, Humanitas University, 20090 Pieve Emanuele, Italy; 3 IRCCS Humanitas Research Hospital, 20089 Rozzano, Italy; 4 Regional Center of Nuclear Medicine, Department of Translational Research and Advanced Technologies in Medicine and Surgery, University of Pisa, 56126 Pisa, Italy; 5 Medical Imaging Center, University of Groningen, University Medical Center Groningen, Groningen, The Netherlands; 6 Human Technopole, Health Data Science Center, 20157 Milan, Italy

Correction to: *Scientific Reports* 10.1038/s41598-022-23752-2, published online 15 November 2022

Martina Sollini and Paola Anna Erba were omitted from the author list in the original version of this Article.

The Author Contributions section now reads:

“L.C. conceived the pipeline, set up the case study, analysed the results, prepared the figures, and wrote the manuscript. M.P. formulated and tuned the pruned tree edit distance, provided the mathematical proofs and the simulation study, and wrote the manuscript. A.R. contributed to implement the patient representation pipeline. M.S. segmented the Prostate Cancer lesions and extracted the radiomic features for all patients in the case study. P.A.E collected the data and enrolled the patients in the clinical study. F.I. supervised the analyses and the conception of the pipeline. L.C., M.P., A.R. and F.I. reviewed and approved the manuscript.”

The original Article and accompanying Supplementary Information file have been corrected.

