# Supplementary material for: Imaging-based representation and stratification of intra-tumor heterogeneity via tree-edit distance
Source: Sci Rep. 2022 Nov 15;12:19607. doi: 10.1038/s41598-022-23752-2 (PMC9666477; doi:10.1038/s41598-022-23752-2)
Supplement: Supplementary file 1 — Supplementary Information. [file 41598_2022_23752_MOESM1_ESM.pdf]

Imaging-based representation and stratification of intra-tumor Heterogeneity via tree-edit distance: Supplementary materials

Lara Cavinato[1,*,+], Matteo Pegoraro[1,+], Alessandra Ragni[1], Martina Sollini[2,3], Paola Anna Erba[4,5], and Francesca Ieva[1,6]

[1]MOX Lab, Department of Mathematics, Politecnico di Milano, Milan, 20133, Italy
[2]Department of Biomedical Sciences, Humanitas University, Pieve Emanuele, 20090, Italy
[3]IRCCS Humanitas Research Hospital, Rozzano, 20089, Italy
[4]Regional Center of Nuclear Medicine, Department of Translational Research and Advanced Technologies in Medicine and Surgery, University of Pisa, 56126, Pisa, Italy
[5]Medical Imaging Center, University of Groningen, University Medical Center Groningen, Groningen, The Netherlands
[6]Human Technopole, Health Data Science Center, Milan, 20157, Italy
[*]lara.cavinato@polimi.it
[+]These authors contributed equally to this work

ABSTRACT

Personalized medicine is the future of medical practice. In oncology, tumor heterogeneity assessment represents a pivotal step for effective treatment planning and prognosis prediction. Despite new procedures for DNA sequencing and analysis, non-invasive methods for tumor characterization are needed to impact on daily routine. On purpose, imaging texture analysis is rapidly scaling, holding the promise to surrogate histopathological assessment of tumor lesions. In this work, we propose a tree-based representation strategy for describing intra-tumor heterogeneity of patients affected by metastatic cancer. We leverage radiomics information extracted from PET/CT imaging and we provide an exhaustive and easily readable summary of the disease spreading. We exploit this novel patient representation to perform cancer subtyping according to hierarchical clustering technique. To this purpose, a new heterogeneity-based distance between trees is defined and applied to a case study of Prostate Cancer (PCa). Clusters interpretation is explored in terms of concordance with severity status, tumor burden and biological characteristics. Results are promising, as the proposed method outperforms current literature approaches. Ultimately, the proposed method draws a general analysis framework that would allow to extract knowledge from daily acquired imaging data of patients and provide insights for effective treatment planning.

1 Patients' personal information summary

Supplementary Tables S1 and S2 summarize the patients' population.

Variable	Mean	Std. dev.	Median	Range
Age	72.09	7.03	71.68	54.88 – 85.24
Total volume	16.41	34.72	3.16	0.22 – 207.70
Gleason Score	7.73	1.03	7.00	5.00 – 9.00
PSA	18.16	70.96	2.66	0.09 – 591.00

Table S 1. Statistical summary of patients' personal information (continuous variables).

2 Distance metrics for trees: brief literature review

In this section we present a high level overview of the literature concerning the comparison between different trees.

Dendrograms are *unlabelled* object which, in our context, may have a different number of leaves and do not hold any a-priori correspondence between the leaves in different objects. The literature dealing with the comparison of dendrograms divide in

Variable		Number of patients (%)
Number of metastases	Oligo (<3)	38 (41.3%)
	Multi (≥3)	54 (58.7%)
	Oligo (<5)	60 (65.22%)
	Multi (≥5)	32 (34.78%)
	Intermediate (3≤n<5)	22 (23.92%)
Gleason Score (dichotomous)	<7	8 (8.7%)
	=7	45 (48.91%)
	>7	31 (33.69%)
	missing	8 (8.7%)
Ongoing therapy	Y	33 (35.87%)
	N	59 (64.13%)
Initial therapy	RP	23 (25%)
	RP+RT	52 (56.52%)
	RT	9 (9.78%)
	missing	8 (8.7%)
PSA (dichotomous)	≤ 1.93	33 (35.87%)
	>1.93	48 (52.17%)
	missing	11 (11.96%)

Table S 2. Statistical summary of patients' personal information (categorical variables).

two macro-areas, including (1) metrics defined for clustering dendrograms and (2) metrics designed for *merge trees*. The first family of metrics mainly deals with *labeled* trees [1, 2] as byproducts of a hierarchical clustering algorithm. We refer to Flesia et al. [3] for an exhaustive review of distance definitions. This kind of metrics are known to be heavily dependent on the graph structure of the dendrograms, like many others [4, 5, 6, 7], leading to limitations when comparing dendrograms with a different number of leaves and lacking theoretical continuity results with respect to dendrogram-associated point clouds [8, 9]. On the other hand, within topological data analysis [10], dendrograms are often referred as a particular case of merge trees, obtained when all the leaves of a merge tree lie at height 0. This allows to transfer merge trees literature to dendrogram analysis. Most of the metrics belonging to this second family, however, share one main drawback, namely the out of reach computational cost, which makes them unsuitable for our application [11, 12, 13]. Besides, the metrics with more performing algorithms [14, 15] still lack the theoretical investigation to assess some practical properties, making them less worthy than others.

3 Building the vertices heights curve

In Figure 1, we explain in details how to build the curve displaying the filtered heights of one tree' vertices. In the plot there are three detailed examples showing how to obtain the curves in Figure 2a. The colors of the curves match the colors of the trees. For each tree and for every $h \in \mathbb{R}$, the value $f(h)$ is equal to the number of vertices (highlighted with dots) which lie above h. For instance consider the blue dendrogram: we obtain a value of 3 until we reach the height where two leaves merge, and then 1 for a small interval of values, and lastly 0 from the height of the root of the blue tree onwards.

4 Dendrograms construction

In this section we present few technical definition that we need in order to describe the dendrogram representation we employ. We describe the procedure in the general case of having a finite metric space (X,d) i.e. a finite set $\{x_1,\dots,x_n\}$ with a metric $d : X \times X \to \mathbb{R}_{\geq 0}$ which is reflexive, symmetric and satisfies the triangular inequality. In our case we work with $\{x_1,\dots,x_n\} \subset \mathbb{R}^n$ and the Euclidean norm.

Definition 1. *A tree structure T is given by a finite set of vertices V_T and set of edges $E_T \subset V_T \times V_T$ which form a connected rooted acyclic graph. The order of a vertex is the number of edges which have that vertex as one of the extremes. Any vertex with an edge connecting it to the root is its child and the root is its father. In this way we recursively define father and children (possibly none) relationships for any vertex on the tree. The vertices with no children are called leaves and are collected in the set L_T, while the set of children of a vertex $x \in V_T$ is called $child(x)$. Similarly, the vertex $father(x)$ is the father of the vertex x.*

The relationship $father > child$ induces a partial order on V_T. The edges E_T are given in the form of ordered couples (a,b) with $a < b$. For any vertex $v \in V_T$, $sub_T(v)$ is the subtree of T rooted in v, that is the tree structure given by the set of vertices

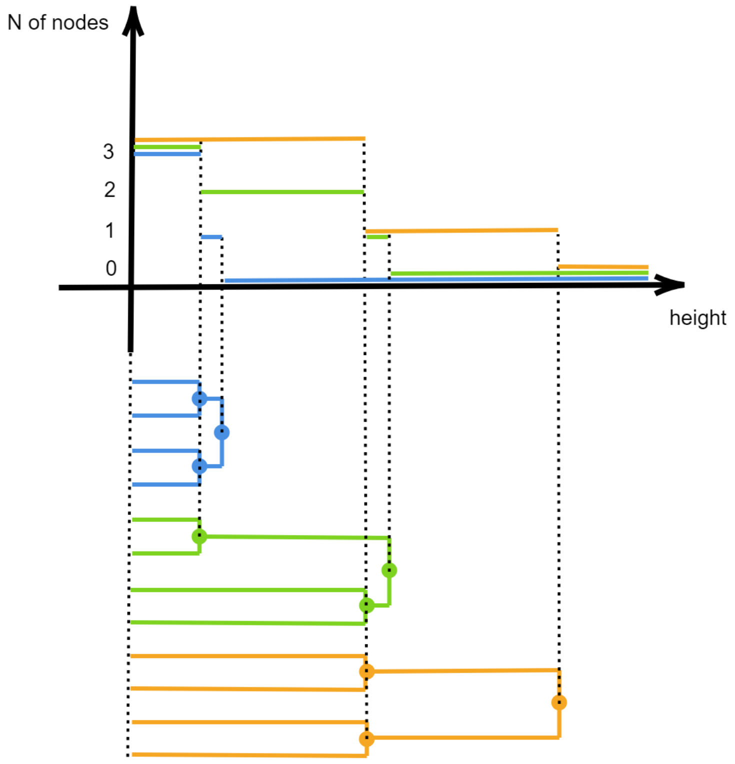

Fig. S 1. Procedure for building the vertices heights curve of three example patient-tree.

$v' \leq v$. *If clear from the context we might omit the subscript* T.

Now, to obtain a dendrogram we need to add some kind of length measure to a tree structure.

Definition 2. *A merge tree* (T, f) *is a finite tree structure* T *coupled with a monotone increasing function (with respect to partial ordering on* V_T*)* $f : V_T \rightarrow \mathbb{R}$*. If* $f(l) = 0$ *for all* $l \in L_T$*, then we say that the merge tree is a dendrogram. The function* f *also defines a weight value for every edge* $e = (v, father(v))$*:* $w_T(e) = f(father(v)) - f(v)$*.*

To build a hierarchical clustering dendrogram T_C from a finite metric space (C, d_C) we proceed as follows. With K we indicate the set of clusters we are considering:

(S0) at the beginning $K = \{\{c\} \mid c \in C\}$, and every $c \in C$ is associated to a leaf $v_c \in V_{T_C}$ with $f(v_c) = 0$;

(S1) consider all the couples of clusters $k_1, k_2 \in K$ and we measure the distance $d(k_1, k_2)$ according to some *linkage*;

(S2) pick $k, k' \in K$ such that $d(k, k') = \min_{k_i \in K; k_1 \neq k_2} d(k_1, k_2)$ and add the vertex $v_{kk'}$ to V_{T_C} with $f(v_{kk'}) = d(k, k')$. Then remove k and k' from K and add $k \cup k'$ to K;

(S3) start again from (S1) unless $K = C$.

The linkage determines the distance $d(k_1, k_2)$ between $k_1, k_2 \subset C$ and the most common examples are:

- single linkage: $d(k_1, k_2) = \min_{c_i \in k_i} d_C(c_1, c_2)$
- complete linkage: $d(k_1, k_2) = \max_{c_i \in k_i} d_C(c_1, c_2)$
- average linkage: $d(k_1, k_2) = (\#k_1 \cdot \#k_2)^{-1} \cdot \sum_{c_i \in k_i} d_C(c_1, c_2)$, where $\#k_i$ is the cardinality of the finite set k_i.
- ward linkage: see [16]

It is well known that single linkage is very sensitive to outliers, while complete linkage is the most conservative choice in term of clustering points together. Average linkage displays a kind of in-between behaviour. For this reason we resorted to average linkage.

5 Continuity Proposition

Having a continuity result of the distance between dendrograms with respect to some metric between point clouds would surely benefit the consistency and the interpretability of the framework: whenever a representation of a datum is employed, looking at how changes in the representation reflect on changes in the initial datum can help both assessing the consistency of the pipeline, and familiarizing with the representation. In our case we are particularly interested in looking at what happens at the distance between dendrograms as two sequences of point clouds get closer and closer. To do so, we introduce the definitions of the Hausdorff and Gromov-Hausdorff metrics between point clouds. We then prove the continuity proposition between Hausdorff distance and the Edit distance, from which it follows the continuity proposition between Gromov-Hausdorff distance and the Edit distance.

Given $C = \{x_1,\dots,x_n\}$ and $C' = \{y_1,\dots,y_m\}$ two point clouds in a metric space (X,d), we can build at least a function $\gamma : C \to C'$ such that $\gamma(x_i)$ is (one of) the closest point(s) to x_i, belonging to the cloud C'. Similarly, we can build $\varphi : C' \to C$ so that $\varphi(y_j)$ is (one of) the closest point(s) to y_j, belonging to the cloud C. The Hausdorff distance between C and C' is given by:

$$d_H(C,C') = \max\{\max_{x\in C} d(x,\gamma(x)),\ \max_{y\in C'} d(y,\varphi(y))\} \tag{1}$$

The distance d_H has been proven to be a metric for the space of all compact subsets of X [17].

Leveraging on the Hausdorff distance, given two compact metric spaces X and Y we define the Gromov-Hausdorff metric as $d_{G-H}(X,Y) := \inf d_H(\gamma(X),\varphi(Y))$ where γ and φ vary over all possible isometries of (respectively) X and Y into another (common) metric space Z [18].

Consider two point clouds in the metric space (X,d), $C = \{x_1,\dots,x_n\}$ and $C' = \{y_1,\dots,y_m\}$ and we consider T_C and $T_{C'}$ the single linkage hierarchical clustering dendrograms obtained from C and C' respectively. In the following, we prove the following result:

Proposition 1. *Given $C = \{x_1,\dots,x_n\}$ and $C' = \{y_1,\dots,y_m\}$ point clouds in a metric space (X,d) and given T_C and $T_{C'}$ single linkage hierarchical clustering dendrograms obtained from C and C' respectively, there is a simplicial complex S and two functions $f : S \to \mathbb{R}$ and $g : S \to \mathbb{R}$ such that the merge tree associated to f (via sublevel set filtration) is isomorphic to T_C, the merge tree associated to g is isomorphic to $T_{C'}$, and $\| f - g \|_\infty \leq 2d_H(C,C')$.*

Proof. Let $\gamma : C \to C'$ and $\varphi : C' \to C$ be the two operators which map a point of a point cloud C' to (one of) the closest point(s) of the other cloud C and viceversa.

Consider the following simplicial complex S. Its 0 simplices are $x_1,\dots,x_n,y_1,\dots,y_m$ and its 1 simplices are all possible edges between 0 simplices, forming a complete graph.

Now we define two functions $f : S \to \mathbb{R}$ and $g : S \to \mathbb{R}$ such that the merge trees T_f and T_g obtained with the lower star filtration from f and g (see [19], Section 2) are isomorphic to T_C and $T_{C'}$.

Define: $f(s) = 0$ for every 0 simplex s. Then for a 1 simplex of the form $e_{ij} = (x_i,x_j)$, we have $f(e_{ij}) = d(x_i,x_j)$. For 1 simplices of the form $e'_{ij} = (y_i,x_j)$ we have $f(e'_{ij}) = d(\varphi(y_i),x_j)$. Lastly, for 1 simplices of the form $e''_{ij} = (y_i,y_j)$ we have $f(e''_{ij}) = d(\varphi(y_i),\varphi(y_j))$. Note that $t \in Im(f)$ iff $t = d(x_i,x_j)$ for some i and j. Clearly, f is a finite set and we can order it: $t_0 = 0 < t_1 < \dots$.

Similarly we define $g(e''_{ij}) = d(y_i,y_j)$, $g(e'_{ij}) = (y_i,\gamma(x_j))$ and $f(e''_{ij}) = (y_i,y_j)$.

Consider now the connected components of the graph $S^f_t := \{s \in S | f(s) \leq t\}$ for $t \in \mathbb{R}$. If $t < 0$, S^f_t is empty. If $t = t_0 = 0$, then all 0 simplices are in S^f_0, plus the 1 simplices of the form (x_i,y_j) such that $\varphi(y_j) = x_i$ and (y_i,y_j) such that $\varphi(y_i) = \varphi(y_j)$. This means that every vertex y_i is connected with exactly one point x_j and with all other y_k such that $\varphi(y_k) = x_j$. That is, there are n path connected components, one for each x_i. Call such components $[x_i]$.

Consider the value $t = t_1 = d(x_i,x_j)$. For every $y \in \varphi^{-1}(x_i)$ and $y' \in \varphi^{-1}(x_j)$, we have $f((y,y')) = f((x_i,y')) = f((y,x_j)) = f((x_i,x_j)) = d(x_i,x_j)$ and so all these 1 simplices get added, when passing from S^f_0 to $S^f_{t_1}$. Moreover, these are the only ones which get added. Which means that we get all possible edges between $[x_i]$ and $[x_j]$ but all others components are left unchanged. And this happens whenever we hit a level $t_k = d(x_i,x_j)$: we add to the simplicial complexes $S^f_{t_k}$ all possible edges between $[x_i]$ and $[x_j]$.

Now, we build the single linkage hierarchical dendogram T_C associated to C, with labels given by $\{\{x_1\},\dots,\{x_n\}\}$, and the merge tree T_f associated to $f : S \to \mathbb{R}$ with labels $\{[x_1],\dots,[x_n]\}$. An internal vertices of T_C indicating the merging of two leaves $\{x_i\}$ and $\{x_j\}$ will be called $\{x_i,x_j\}$, and similarly a vertex called $\{x_i,x_j,x_k\}$ indicates that the leaves of the subtree rooted in that vertex are $\{x_i\}$, $\{x_j\}$ and $\{x_k\}$. In the same fashion, an internal vertex of T_f where to components $[x_i]$ and $[x_j]$ merge is named $[x_i]\bigcup[x_j]$. A vertex called $[x_i]\bigcup[x_j]\bigcup[x_k]$ is associated to the origin of the connected component $[x_i]\bigcup[x_j]\bigcup[x_k]$.

Thus, we can define a map $\eta : V_{T_C} \to V_{T_f}$ induced by $\eta(x_i) = [x_i]$ and $\eta(\{x_i,x_j,x_k\}) = [x_i]\bigcup[x_j]\bigcup[x_k]$ which is an isomorphism of merge trees. An analogous proof yields the isomorphism between $T_{C'}$ and T_g.

To conclude the proof it is enough to notice that: $||f-g||_\infty \leq 2\varepsilon$ with $\varepsilon = d_H(C,C')$. In fact, for vertices s: $f(s) = g(s) = 0$. For an edge e, we have the following possibilities:

- $e = (x_i,x_j)$: $|f(e)-g(e)| = |d(x_i,x_j) - d(\gamma(x_i),\gamma(x_j))|$. We have $d(x_i,\gamma(x_i)) \leq \varepsilon$, $d(x_i,x_j) \leq d(\gamma(x_i),\gamma(x_j)) + 2\varepsilon$ and $d(\gamma(x_i),\gamma(x_j)) \leq d(x_i,x_j) + 2\varepsilon$; which, together, give $|f(e)-g(e)| \leq 2\varepsilon$.
- $e = (y_i,y_j)$: $|f(e)-g(e)| = |d(\varphi(y_i),\varphi(y_j)) - d(y_i,y_j)|$; reasoning as above we obtain $|f(e)-g(e)| \leq 2\varepsilon$
- $e = (x_i,y_j)$: $|f(e)-g(e)| = |d(x_i,\varphi(y_j)) - d(\gamma(x_i),x_j)|$. Again in the same fashion we have: $d(x_i,\varphi(y_j)) \leq d(x_i,y_j) + d(y_j,\varphi(y_j)) \leq d(x_i,\gamma(x_i)) + d(\gamma(x_i),x_j) + d(y_j,\varphi(y_j)) \leq d(\gamma(x_i),x_j) + 2\varepsilon$. Which entails $|f(e)-g(e)| \leq 2\varepsilon$

□

Corollary 1. *Given $C = \{x_1,\dots,x_n\}$ and $C' = \{y_1,\dots,y_m\}$ point clouds in (X,d) metric space, and given T_C and $T_{C'}$ the single linkage hierarchical clustering dendrograms obtained from C and C' respectively, we have $d_E(T_C,T_{C'}) \leq 4(n+m)d_H(C,C')$.*

Proof. We apply Proposition 1 and then we are in the position to use Theorem 1 in [19] to obtain that $d_E(T_f,T_g) \leq 2(2d_H(C,C'))\cdot(n+m)$. □

With the above results, we can prove a last corollary involving the Gromov-Hausdorff distance between compact metric spaces.

Corollary 2. *Given two finite metric spaces $C = \{x_1,\dots,x_n\}$ and $C' = \{y_1,\dots,y_m\}$ and given T_C and $T_{C'}$ the single linkage hierarchical clustering dendrograms obtained from C and C' respectively, we have $d_E(T_C,T_{C'}) \leq 4(n+m)d_{G-H}(C,C')$.*

Proof. We apply Proposition 1 and Corollary 1 on the images $\gamma(X)$ and $\varphi(Y)$ for every $\gamma : X \to Z$, $\varphi : Y \to Z$ isometries, and for every Z metric space. □

6 Proof about d_P^μ being a metric

We prove the following proposition.

Proposition 2. *If there is $M > 0$ such that for every $m \leq M$, $\mu([0,m]) > 0$ the d_μ^P is a metric.*

Proof. □

Suppose $d_P^\mu(T,T') = 0$. Let $m = \min\{\min_{e\in E_T} w_T(e),\ \min_{e'\in E_{T'}} w_{T'}(e')\}$; then for any $\varepsilon \in [0,m)$, $P_\varepsilon(T) = T$ and $P_\varepsilon(T') = T'$. If $d_E(T,T') > 0$, since $\mu([0,m)) > 0$, then:

$$0 < \int_{[0,m)} d_E(P_\varepsilon(T),P_\varepsilon(T'))d\mu(\varepsilon) \leq d_P^\mu(T,T') = 0$$

which is absurd. But then $d_E(T,T') = 0$ and so $T = T'$.
Symmetry is obvious.
The triangle inequality holds for d_E and so

$$d_E(P_\varepsilon(T),P_\varepsilon(T')) \leq d_E(P_\varepsilon(T),P_\varepsilon(T'')) + d_E(P_\varepsilon(T''),P_\varepsilon(T'))$$

The linearity of the integral then entails $d_P^\mu(T,T') \leq d_P^\mu(T,T'') + d_P^\mu({}''T,T')$.

7 Heterogeneity-based Simulation for d_μ^P

In this section, we test the metric d_μ^P and the whole pipeline employed in the case study of the main manuscript in a supervised - in a broad sense - and easier setting. In particular, the aim of this simulation is to showcase the differences between d_E and d_μ^P and to which extent d_μ^P captures heterogeneity in a point cloud.

We generate point clouds in $\mathbb{R}^2$ according to two generating processes. The size n_1^i of the i-th point cloud of the first group is sampled uniformly from $[2,20]\bigcap\mathbb{Z}$ and then a sample of size $(n_1^i,2)$ is taken from a normal distribution $\mathcal{N}(0,\sigma_1)$, with $\sigma_1 = 1$. Similarly, the j-th point cloud of the second group has cardinality n_2^j sampled uniformly from $[2,10]\bigcap\mathbb{Z}$, and the cloud itself is taken as a sample of size $(n_2^j,2)$ distributed according to $\mathcal{N}(0,\sigma_2)$, with $\sigma_2 = 2$. The data set of point clouds contains 50 clouds of the first group and 50 of the second group.

From the data-generating processes it is clear that the sources of variability between the two groups arise potentially from the different cardinalities of the point clouds and variance within each cloud. We want to show that, while the metric d_E is susceptible to both kind of variability, d_μ^P, with an appropriately chosen measure μ, can mitigate the variability coming from higher cardinalities in the clouds sampled according to the first process. In particular, group 1 is expected to display a lower level of heterogeneity within each point cloud and thus those trees, for our purposes, should be regarded as more similar between each other compared to the other trees. The second group instead may not display a clear clustering structure, in fact, despite exhibiting a common level of heterogeneity, the different number of leaves and the different merging structure at the level of very heterogeneous leaves could prevent all such dendrograms to form a recognizable cluster - or, equivalently, could give birth to a cluster with higher dispersion.

Following the pipeline presented in the main manuscript, we extract average linkage hierarchical clustering dendrograms from the set of point clouds and take pairwise distances both with d_E and d_μ^P. Examples of dendrograms belonging to the first and second groups can be found, respectively, in Fig. S3b and S3c. We select μ as in the main manuscript, Section 4.3.2, with the final choice being a Beta distribution with parameters $a = 2$, $b = 8$, as shown in Fig. S2a. The two matrices are reported in Fig. S2, with data being ordered according to the two groups: the first 50 point clouds belong to the first group, and the following 50 to the second. By visual inspection of Fig. S2b and S2c we can clearly see that d_E sees very little structure in the data, because of the two sources of variability (cardinality and variance) mixing up and preventing d_E to discriminate between group 1 and 2. Instead d_μ^P recognizes a clear and pronounced cluster made by point clouds from group 1 plus, potentially, some other point clouds belonging to group 2. The rest of the point clouds of group 2 still show some agglomerative structure, but less evident. The matrix in Fig. S2d shows the pointwise differences between the values obtained with d_E and d_μ^P, highlighting how the different behaviour of the two metrics concentrates on the data belonging to the first group.

To get more insights into the clustering structures expressed by d_E and d_μ^P we extract the hierarchical clustering dendrograms with average linkage from the two matrices. These dendrograms are reported in Fig. S3a. The leftmost tree is obtained from d_E and the central from d_μ^P. To better compare the clustering structures we remove from this last dendrogram the outlier ($v53$), obtaining the rightmost tree.

Visual inspection of the dendrograms in Fig. S3a reveals a two-clusters structure in both metric spaces, with this structure being much more recognizable in the metric space induced by d_μ^P. In particular, the rightmost dendrogram shows a very cohesive and compact cluster, with very low internal variability, which is absent in the leftmost tree. The other cluster of the same tree, instead, displays a much higher level of variability.

Now we show that this clustering structure reflects the group structure that generated our data. We cut the rightmost tree to obtain two clusters. Then, for each cluster, we aggregate the points contained in the data of such cluster and we estimate the marginal densities from the obtained samples. The results of this estimation pipeline is showcased in Fig. S4. We see that we retrieve the two distributions which we used to generate the components of the point clouds of the two groups.

This is precisely the behaviour we aimed to achieve: being insensitive to the cardinality of small homogeneous features, while still being sensitive to cardinalities and merging structures characterized by high heterogeneity.

8 Additional plots for clustering interpretation

Supplementary Fig. S5 integrates the results, in terms of cluster characterization.

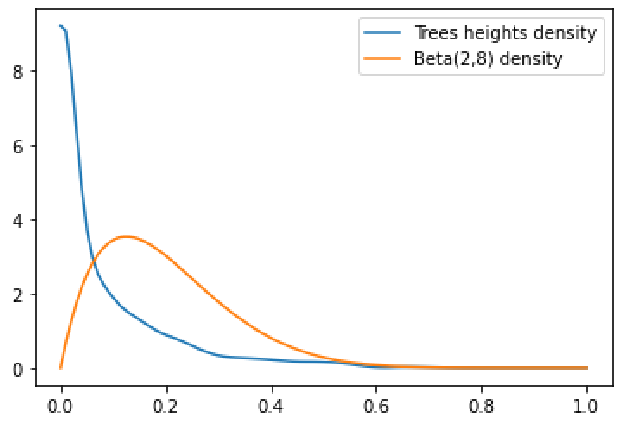

(a) Density of vertices heights from trees in the simulation data, along with the chosen Beta distribution, which has parameters $a = 2$ and $b = 8$.

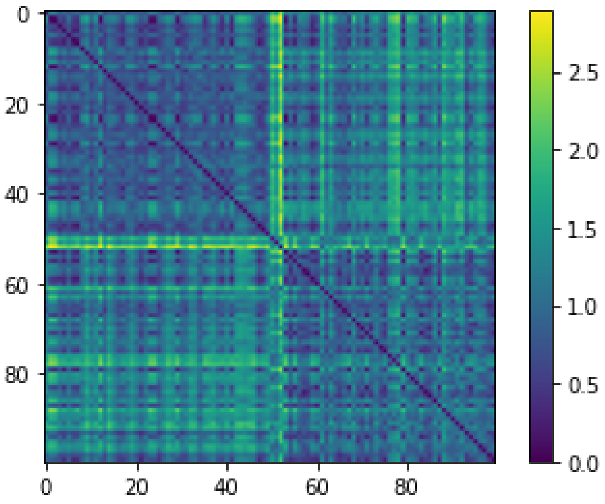

(b) Matrix of pairwise distances obtained with d_E.

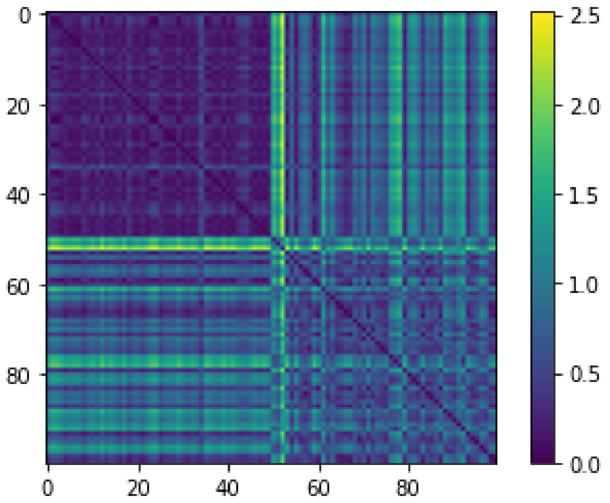

(c) Matrix of pairwise distances obtained with d^P_μ.

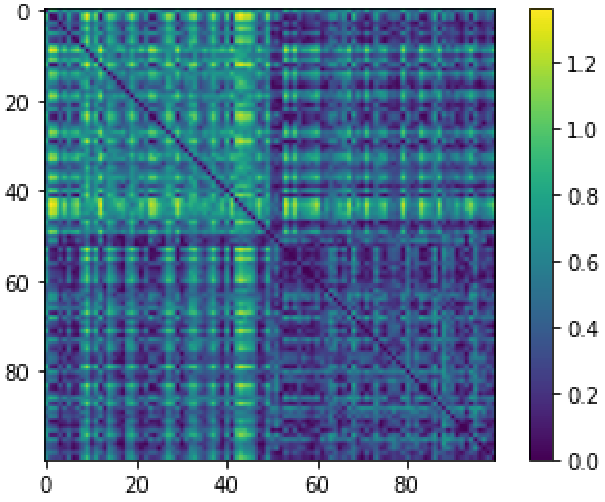

(d) Absolute differences between the matrix obtained with d_E and d^P_μ.

Fig. S 2. The plot in the left upper corner is used to fix μ in the case study of Section 7, according to the procedure detailed in Figure 7 of the manuscript; the other figures show the pairwise distance matrices obtained in the case study of Section 7.

References

1. Robinson, D. F. & Foulds, L. R. Comparison of weighted labelled trees. In *Combinatorial mathematics VI*, 119–126 (Springer, 1979).
2. Billera, L. J., Holmes, S. P. & Vogtmann, K. Geometry of the space of phylogenetic trees. *Adv. Appl. Math.* **27**, 733–767 (2001).
3. Flesia, A. Unsupervised classification of tree structured objects. In *BIOMAT 2008*, 280–299 (World Scientific, 2009).
4. Poon, A. F. *et al.* Mapping the shapes of phylogenetic trees from human and zoonotic rna viruses. *PLoS one* **8**, e78122 (2013).
5. Colijn, C. & Plazzotta, G. A metric on phylogenetic tree shapes. *Syst. Biol.* **67**, 113–126 (2018).
6. Lewitus, E. & Morlon, H. Characterizing and comparing phylogenies from their laplacian spectrum. *Syst. biology* **65**, 495–507 (2016).

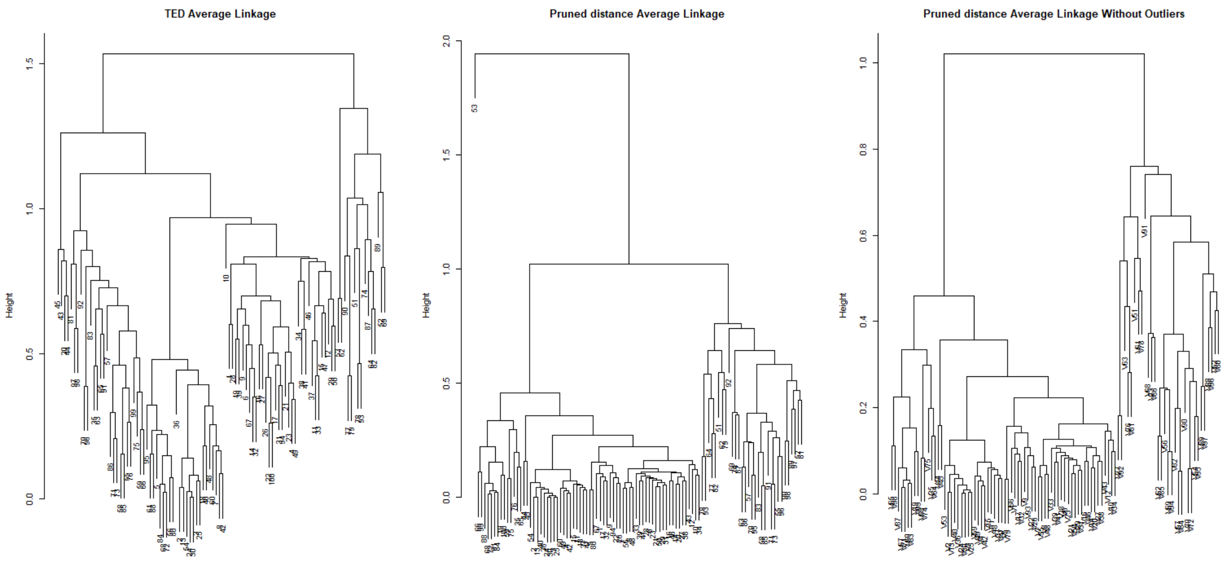

(a) Hierarchical Clustering with average linkage of the pairwise distance matrices respectively obtained from d_E, d_μ^P and d_μ^P but without the outlier represented by vertex 53 in the central dendrogram.

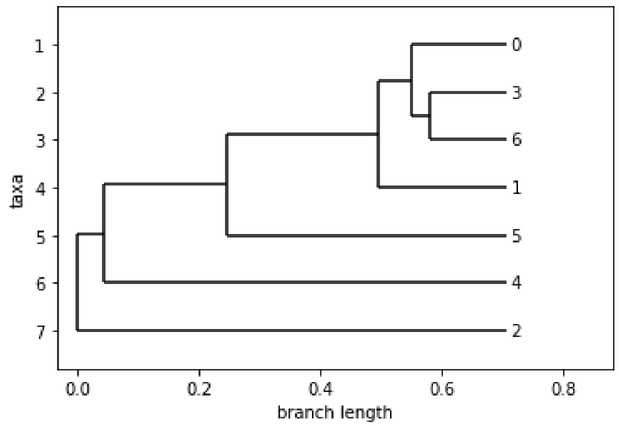

(b) The outlier identified by the hierarchical clustering with average linkage of the matrix induced by d_μ^P.

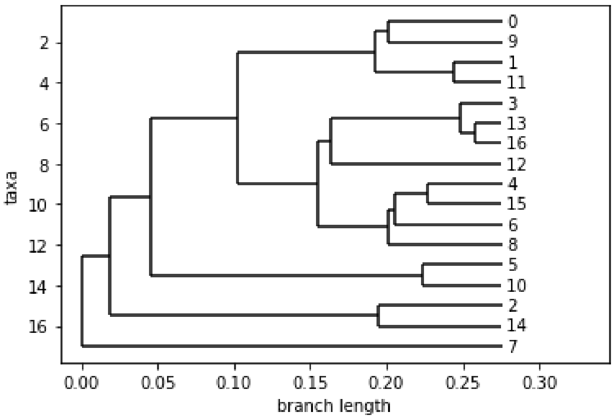

(c) A randomly chosen dendrogram belonging to group 2. The difference in terms of heteogeneity between leaves and number of leaves, with the dendrogram in Fig. 3b is evident.

Fig. S 3. Cluster analysis of pairwise distance matrices obtained in the case study of Section 7.

7. Kim, J., Rosenberg, N. A. & Palacios, J. A. Distance metrics for ranked evolutionary trees. *Proc. Natl. Acad. Sci.* **117**, 28876–28886 (2020).

8. Smith, M. R. Bayesian and parsimony approaches reconstruct informative trees from simulated morphological datasets. *Biol. letters* **15**, 20180632 (2019).

9. Smith, M. R. Information theoretic generalized robinson–foulds metrics for comparing phylogenetic trees. *Bioinformatics* **36**, 5007–5013 (2020).

10. Edelsbrunner, H. & Harer, J. Persistent homology—a survey. In *Surveys on discrete and computational geometry*, vol. 453 of *Contemporary Mathematics*, 257–282 (American Mathematical Society, Providence, RI, 2008).

11. Beketayev, K., Yeliussizov, D., Morozov, D., Weber, G. H. & Hamann, B. Measuring the distance between merge trees. In *Topological Methods in Data Analysis and Visualization*, 151–166 (Springer International Publishing, Cham, 2014).

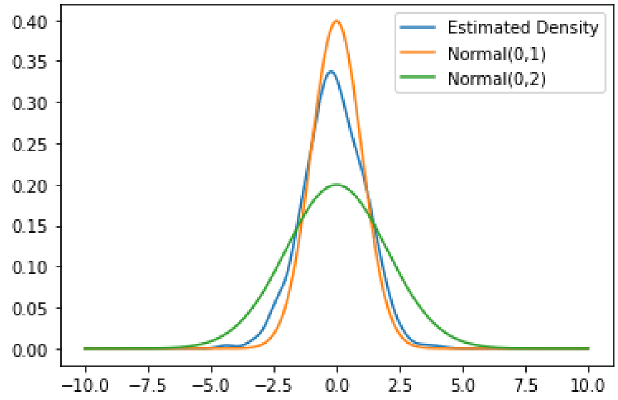

(a) **Estimated density of the first component of the data in the first cluster identified by d_μ^P, versus the densities generating the samples two groups.**

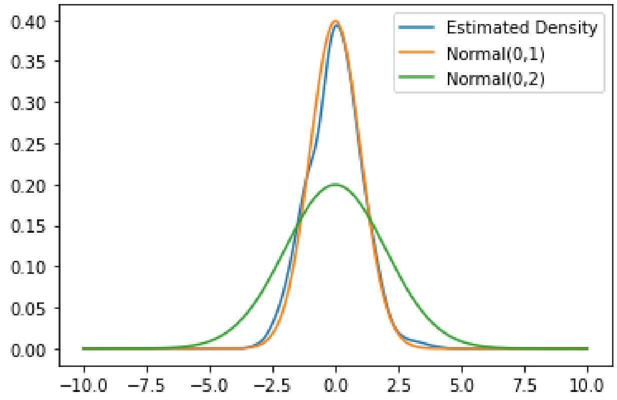

(b) **Estimated density of the second component of the data in the first cluster identified by d_μ^P, versus the densities generating the samples two groups.**

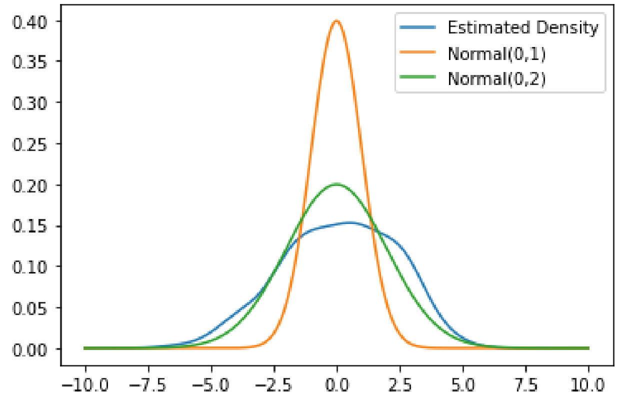

(c) **Estimated density of the first component of the data in the second cluster identified by d_μ^P, versus the densities generating the samples two groups.**

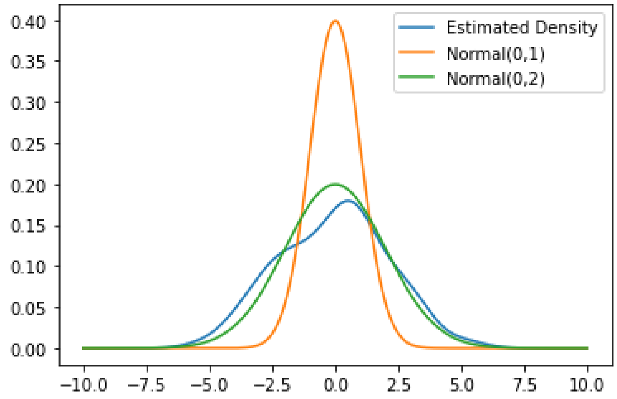

(d) **Estimated density of the second component of the data in the second cluster identified by d_μ^P, versus the densities generating the samples two groups.**

Fig. S 4. Densities estimated through the aggregation of the data collected in the two clusters identified by d_μ^P.

12. Bauer, U., Landi, C. & Mémoli, F. The reeb graph edit distance is universal. *Foundations Comput. Math.* **21**, 1441–1464 (2021).

13. Cardona, R., Curry, J., Lam, T. & Lesnick, M. The universal lp-metric on merge trees. *arXiv preprint arXiv:2112.12165* (2021).

14. Pont, M., Vidal, J., Delon, J. & Tierny, J. Wasserstein distances, geodesics and barycenters of merge trees. *IEEE Transactions on Vis. Comput. Graph.* **28**, 291–301 (2021).

15. Sridharamurthy, R., Masood, T. B., Kamakshidasan, A. & Natarajan, V. Edit distance between merge trees. *IEEE Transactions on Vis. Comput. Graph.* **26**, 1518–1531 (2020).

16. Jr., J. H. W. Hierarchical grouping to optimize an objective function. *J. Am. Stat. Assoc.* **58**, 236–244 (1963).

17. Rockafellar, R. T. & Wets, R. J.-B. *Variational analysis*, vol. 317 (Springer Science & Business Media, 2009).

18. Burago, D., Burago, Y. & Ivanov, S. *A course in metric geometry*, vol. 33 (American Mathematical Society, 2022).

19. Pegoraro, M. & Secchi, P. Functional data representation with merge trees. *arXiv preprint arXiv:2108.13147* (2021).

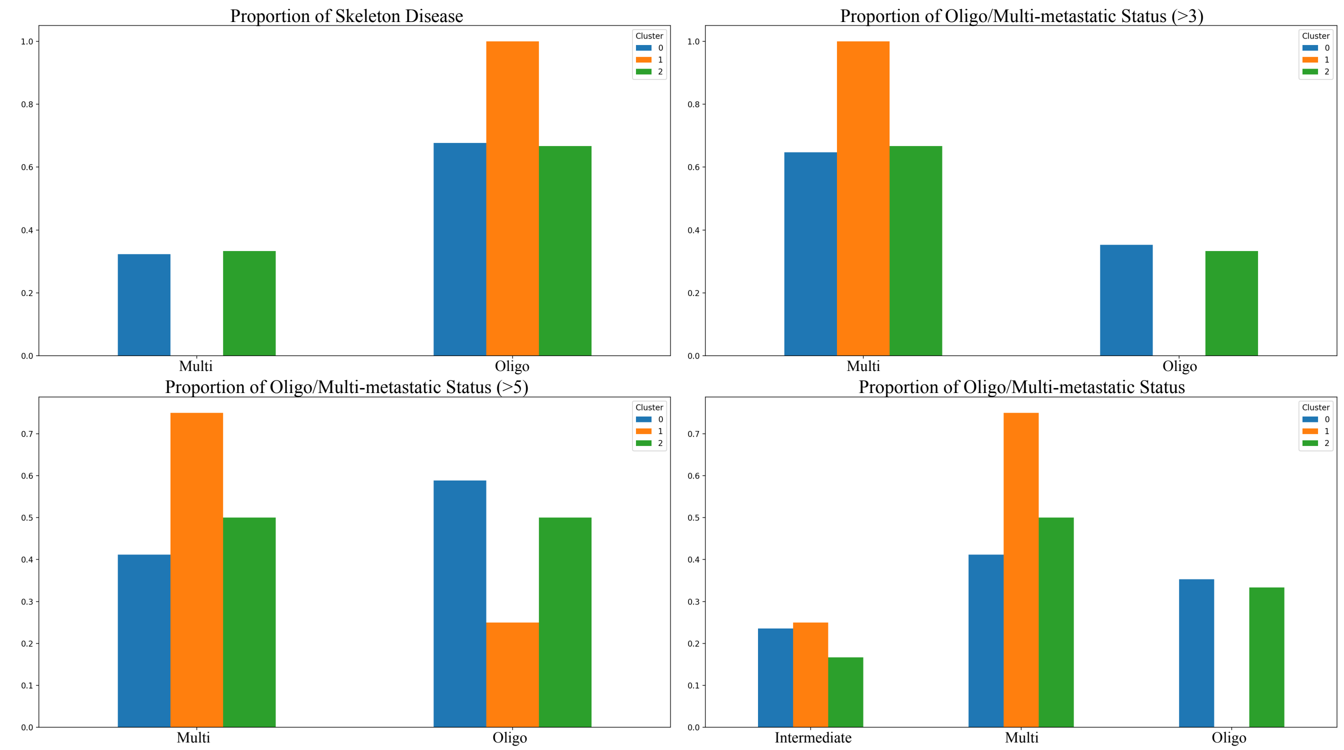

Fig. S 5. Results of clustering characterization: the proportion of skeleton disease and of the oligo/multi-metastatic status as devised by the two clinical cut-offs (3 and 5 lesions) are plotted per each of the three group.
